# An Overview of the Cardiorenal Protective Mechanisms of SGLT2 Inhibitors

**DOI:** 10.3390/ijms23073651

**Published:** 2022-03-26

**Authors:** Teresa Salvatore, Raffaele Galiero, Alfredo Caturano, Luca Rinaldi, Anna Di Martino, Gaetana Albanese, Jessica Di Salvo, Raffaella Epifani, Raffaele Marfella, Giovanni Docimo, Miriam Lettieri, Celestino Sardu, Ferdinando Carlo Sasso

**Affiliations:** 1 Department of Precision Medicine, University of Campania Luigi Vanvitelli, Via De Crecchio 7, 80138 Naples, Italy; teresa.salvatore@unicampania.it; 2 Department of Advanced Medical and Surgical Sciences, University of Campania Luigi Vanvitelli, Piazza Luigi Miraglia 2, 80138 Naples, Italy; raffaele.galiero@unicampania.it (R.G.); alfredo.caturano@unicampania.it (A.C.); luca.rinaldi@unicampania.it (L.R.); annadimarti@alice.it (A.D.M.); gaetanaalbanese@hotmail.it (G.A.); jessydisalvo@hotmail.it (J.D.S.); ellaphane@gmail.com (R.E.); raffaele.marfella@unicampania.it (R.M.); giovanni.docimo@unicampania.it (G.D.); celestino.sardu@unicampania.it (C.S.); 3 Mediterrannea Cardiocentro, 80122 Napoli, Italy; 4 Division of Cardiovascular Sciences, Faculty of Biology, Medicine and Health, The University of Manchester, 3.31 Core Technology Facility, 46 Grafton Street, Manchester M13 9NT, UK; miriam.lettieri@manchester.ac.uk

**Keywords:** type 2 diabetes mellitus, gliflozins, cardiovascular disease, diabetic kidney disease, cardiorenal protection, cardiorenal syndrome

## Abstract

Sodium-glucose co-transporter 2 (SGLT2) inhibitors block glucose reabsorption in the renal proximal tubule, an insulin-independent mechanism that plays a critical role in glycemic regulation in diabetes. In addition to their glucose-lowering effects, SGLT2 inhibitors prevent both renal damage and the onset of chronic kidney disease and cardiovascular events, in particular heart failure with both reduced and preserved ejection fraction. These unexpected benefits prompted changes in treatment guidelines and scientific interest in the underlying mechanisms. Aside from the target effects of SGLT2 inhibition, a wide spectrum of beneficial actions is described for the kidney and the heart, even though the cardiac tissue does not express SGLT2 channels. Correction of cardiorenal risk factors, metabolic adjustments ameliorating myocardial substrate utilization, and optimization of ventricular loading conditions through effects on diuresis, natriuresis, and vascular function appear to be the main underlying mechanisms for the observed cardiorenal protection. Additional clinical advantages associated with using SGLT2 inhibitors are antifibrotic effects due to correction of inflammation and oxidative stress, modulation of mitochondrial function, and autophagy. Much research is required to understand the numerous and complex pathways involved in SGLT2 inhibition. This review summarizes the current known mechanisms of SGLT2-mediated cardiorenal protection.

## 1. Introduction

The sodium-glucose co-transporter 2 or sodium-glucose linked transporter 2 (SGLT2)-inhibitors (SGLT2-Is), also named gliflozins, are a relatively novel class of oral agents used in the treatment of type 2 diabetes mellitus (T2DM). Their mechanism of action involves the inhibition of SGLT2 channels located in the renal proximal convoluted tubule, which is responsible for approximately 90% of filtered glucose reabsorption [1].

SGLT2-Is reduce the renal threshold for glucose excretion from about 10 mmol/L (180 mg/dL) to 2.2 mmol/L (40 mg/dL) [2]. The consequent increase in urinary glucose elimination lowers blood glucose levels. This reduces glucotoxicity and improves β-cell function and whole-body insulin sensitivity in the same way other anti-hyperglycemic drugs do [3].

Phlorizin, the ancestor of SGLT inhibitors, was isolated in 1835 from apple tree root bark, with its glycosuric properties being identified a century later [4,5]. In the past decade, several SGLT2-Is have been developed as derivatives of this drug, including dapagliflozin, canagliflozin, empagliflozin, ertugliflozin, ipragliflozin, sotagliflozin, remogliflozin etabonate, luseogliflozin, and tofogliflozin. In addition to their structural differences, these compounds exhibit variable half-life and selectivity for SGLT2 co-transporters. Only the first four were currently approved for clinical use in Europe.

The protective effects of SGLT2-Is in controlling glycemia cardiovascular (CV) outcomes are known, even during acute events [6,7,8,9,10]. Therefore, early clinical studies on SGLT2-Is focused on ameliorating glucose plasma levels and other diabetes-related effects. However, the CV benefits observed in several studies aroused great interest in their therapeutic advantages, which go beyond the glycemic control and prompted the design of a series of CV outcome trials (CVOTs) conducted during the past 6 years. Unexpectedly, for the first time in the history of diabetology, these drugs demonstrated a compelling reduced risk of cardiovascular disease (CVD) in general, with a more prominent effect on CV death and hospitalization for heart failure. They also showed a reduced risk of chronic kidney disease (CKD), including in non-diabetic subjects.

The research undertaken so far to identify the mechanisms of action of SGLT2-Is has led to impressive results. The lack of a consistent glucose dependency of cardiorenal protection, with no significant difference between subjects with or without T2DM and across the level of glucose control within T2DM patients, suggested a prevalent role for non-glucose mediated pathways [11]. In the past decade, recent observations based mainly on preclinical studies have revealed a surprisingly wide variety of beneficial actions. On the kidney, SGLT2-Is works by restoring the aberrant tubule-glomerular feedback (TGF) and reducing intra-glomerular pressure. On the heart, they modulate the cardiac sodium–hydrogen exchangers. Other effects are systemic metabolic and hemodynamic adjustments, attenuation of mitochondrial dysfunction, oxidative stress and inflammation, stimulation of autophagy, and more [12].

**Figure 1:**
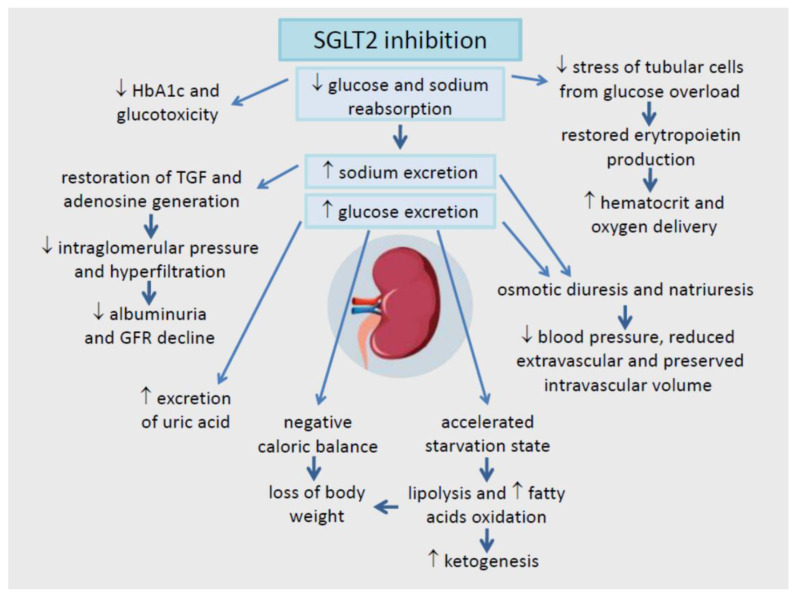
Summary of cardiorenal protective effects driven by inhibition of SGLT2.

**Figure 2:**
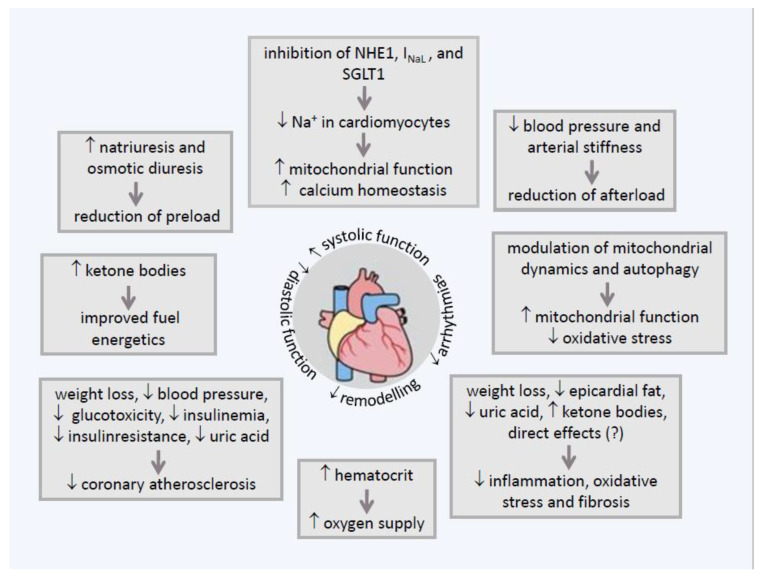
Overview of the main pleiotropic effects of SGLT2-inhibitors on the heart.

In this review, the authors have gathered the extensive scientific research published so far and summarized the current state of knowledge of the mechanisms of SGLT2 inhibition in cardiorenal protection. Figure 1 and Figure 2.

## 2. The Anti-Hyperglycemic Effect of SGLT2-Is

SGLT2-Is are an effective class of agents for the management of hyperglycemia of T2DM, achieving reductions in glycosylated hemoglobin (HbA1c) of 7–10 mmol/mol (0.6–0.9%) when compared with placebo [13]. Based on the inhibition of glucose reabsorption from pre-urine, the glucose-lowering effect of SGLT2-Is only occurs in a hyperglycemic/glycosuric state and, compared to other therapeutic strategies for diabetes, through an action independent of insulin, they are not affected by the deterioration of β-cell function or insulin sensitivity, nor associated with a hypoglycemic risk, even in non-diabetic subjects [14].

The anti-hyperglycemic efficacy of these drugs decreases progressively as blood glucose concentration falls, such that in well-controlled diabetic patients only light glycosuria is detected. Similarly, gliflozins administered to nondiabetic normoglycemic individuals induce small amounts of urinary glucose excretion and negligible lowering of circulating glucose levels [15]. This glycemic pattern is consistent with the observation that hypoglycemia with SGLT2-Is, at least when used as monotherapy, is uncommon. 

The other factor influencing the extent of the glucose-lowering effect is the glomerular filtration rate (GFR); hence the higher the GFR, the greater is the absolute amount of glucose excreted in the urine. Conversely, the lower the GFR, the smaller is the glycosuria. Anyway, it was found that the inhibition towards renal glucose reabsorption was constant across all levels of renal function, indicating that, regardless of GFR, inhibition of glucose reabsorption might reach its maximum [16]. Accordingly, an average of 0.79% reduction in HbA1c is obtained in normal renal function, 0.3–0.4% in the estimated GFR (eGFR) range of 30–59 mL/min/1.73 m^2^, until the total ineffectiveness when the parameter is less than 30 mL/min/1.73 m^2^ [17].

Administration of SGLT2-Is induces adaptations of hormones involved in glucose metabolism, possibly to compensate for increased urinary excretion of glucose, consisting of a decrease in plasma insulin and an increase in plasma glucagon levels with reduced insulin to glucagon ratio [15,18]. It has recently been reported that SGLT2-Is increase pre-proglucagon gene expression by acting directly upon pancreatic α-cells [19].

A substantial increase in the levels of glucagon-like peptide 1 (GLP-1), one of the brain-gut peptides promoting the glucose-dependent release of insulin and the inhibition of glucagon secretion, has been observed in T2DM patients treated with sotagliflozin, an agent that inhibits both SGLT-1 in the gastro-intestinal tract and SGLT-2 in the kidney. This effect likely depends on delayed SGLT1-mediated intestinal glucose absorption and may contribute to controlling blood glucose and body weight exerted by this drug [20].

### 2.1. The Durability of SGLT2-Is

The durability of a glucose-lowering agent is the capacity to maintain HbA1c control over time and to postpone the need for intensification of antidiabetic treatment. The progressive loss of β-cell function characterizing the natural history of T2DM is a main causal factor for an impending deterioration of glycemic control. An eventual increase in insulin resistance caused by aging or weight gain, or other pathological states, may associate.

Only a few studies have evaluated the effects of SGLT2-Is durability. Some authors calculated that the time of HbA1c neutrality (i.e., return to baseline values) was 6–8 years for canagliflozin and full dosage of empagliflozin, a result similar to that for the maximum dose of rosiglitazone and pioglitazone, but superior to that for metformin (5 years) and the dypeptidyl peptidase IV (DPP-4) inhibitors and sulfonylurea classes medications (3–4 years) [21]. A recently published post-hoc analysis of the EMPA-REG OUTCOME trial documented the durability of empagliflozin based on a markedly delayed time to insulin initiation and an early but sustained reduction of daily insulin requirements in patients already treated with insulin [22]. This last effect was reported even with other SGLT2-Is by a previous meta-analysis of nine early-phase RCTs [23].

Clinical studies confirmed the durability of glycometabolic effects for gliflozin at a comparable extent with respect to RCTs and of greater magnitude than those obtained with inhibitors of DPP4 [24,25,26].

The amelioration by SGLT2 inhibition of either β-cell function or peripheral insulin sensitivity, or both, may explain the postponement/reduction of insulin requirement. These effects, demonstrated in both experimental models and human studies, could result from correction of glucotoxicity, reductions in body weight and liver fat, lowering of tissue inflammation, modulation of adipocyte-derived cytokine production, and increase of β-cell proliferation [18,22,27,28,29,30]. Among these hypothesized factors, it has been calculated that a long-term weight loss of at least 2% is significantly associated with maintaining the anti-diabetic therapeutic efficacy [31]. Interestingly, in this study, gliflozins exhibited a lower coefficient of failure than other commonly used glucose-lowering medications.

### 2.2. The Combination Therapy with Other Anti-Hyperglycemic Agents

The use of SGLT2-Is in combination with other specific anti-diabetic drugs has been the subject of various studies.

The association with the GLP-1 receptor agonists (GLP-1RAs) could prevent the decrease in plasma insulin and the increase in plasma glucagon induced by gliflozins and obtain a greater decline in both circulating blood glucose and body weight [32]. In an RCT evaluating T2DM inadequately controlled with metformin, the co-initiation of dapagliflozin with exenatide injections once weekly, in comparison to either drug given as monotherapy, determined after 28 weeks of therapy, a greater reduction of body weight in the exenatide plus dapagliflozin group and a −2.0% change from baseline HbA1c with respect to −1.6% in the exenatide group and −1.4% in the dapagliflozin group [33]. In a later meta-analysis of RCTs, administration of the maximum dose of a GLP-1RA on top of an SGLT2-I compared to SGLT2-I alone resulted in a significant decrease of HbA1c by 0.91%, body weight by 1.95 kg, and systolic blood pressure (BP) by 3.64 mmHg. These advantages paid the price of an increased risk for any hypoglycemia and gastrointestinal adverse events [34].

In a systematic review and meta-analysis of 14 RCTs involving 4828 treatment naïve or metformin-treated patients, the combination therapy of an SGLT2-I with a DPP-4 inhibitor significantly decreased HbA1c (−0.71%), body weight (−2.05 kg), and systolic BP (−5.90 mm Hg), but increased total cholesterol of 3.24%, HDL-C of 6.15% and LDL-C of 2.55% and the risk of genital infections, when compared to DPP-4 inhibition monotherapy [35]. In addition, it was shown that low doses of the SGLT2-I might be a better choice when combination therapy is required being associated slightly but significantly with more reduction in body weight and diastolic BP and with less increase in TC and LDL-C. In an indirect meta-analysis, SGLT2-Is achieved better glycemic control and greater weight reduction than DPP4 inhibitors, without increasing the risk of hypoglycemia in patients with T2DM inadequately controlled with insulin [36].

A recent review summarized the advantages resulting from clinical trials and meta-analyses of SGLT2-Is plus insulin therapy as a treatment regimen for T2DM patients. Compared with placebo, SGLT2 inhibitors plus insulin therapy could significantly decrease HbA1c, the daily insulin dose requirement, and body weight. Other benefits are improvements in insulin resistance and β-cell function and reduction in blood pressure and visceral adipose tissue volume. Against these desirable effects, an increased risk of genital infection, euglycemic diabetic ketoacidosis, and volume depletion have been reported. Overall, the combination therapy appears to be an effective therapeutic option for insulin-treated T2DM patients, provided that careful monitoring of adverse events is ensured [37].

In a meta-analysis of ten eligible placebo-controlled trials, the combination of SGLT2-Is with insulin treatment is beneficial in patients with T1DM compared with placebo, providing reduction of HbA1c, mean amplitude of glucose excursions, body weight, and insulin requirements, without increasing the risk of hypoglycemia but increasing that of genital infections and diabetic ketoacidosis. The authors concluded that although adverse events occur, the available data provide evidence that the combination of SGLT inhibitors with insulin treatment is beneficial in patients with T1DM [38].

Combined therapy with SGT2-Is plus pioglitazone would theoretically provide several advantages. The strong diuretic effect of gliflozins could mitigate the fluid retention induced by pioglitazone, particularly in patients at risk for developing HF. The body fat and weight gain observed with the thiazolidinedione might also be attenuated with the addition of an SGLT2-I agent [39]. For the moment, we only know that compared to pioglitazone alone, the addition of an SGLT2-I may improve glycemic control and reduce both body weight and BP, but increase genital tract infections [40].

### 2.3. The Adverse Effects of SGLT2-I Therapy

In addition to the largely demonstrated protective effects, SGLT2-Is present some side effects, listed as majors and minors, also reported by analysis from clinical trials [41]. Side effects vary (e.g., hypoglycemia, bladder cancer, bone fracture, Fournier’s gangrene, legs amputation, etc.). However, as observed from several authors, the primary and well-recognized are euglycemic diabetic ketoacidosis (DKA) and urinary tract infection (UTIs) [41]. DKA is reported with an incidence rate varying from 0.16 to 0.76 events per 1000 patient-year and recognized associated risk factors are malnutrition, infectious, weight loss, vomiting, or imbalance of insulin doses [42]. The etiopathogenesis is still not fully clear. Some authors observe that SGLT-Is, through direct and indirect effects, stimulate lipolysis, liver ketogenesis, and an insulin production reduction, thus increasing ketone storage and ketonemia, and consequently the risk of DKA [43]. Moreover, it seems that increased renal reabsorption of ketones and the hypovolemia induced by SGLT-Is could increase this risk.

The increased risk of UTIs is the other main side effect, as reported in several clinical trials [41]. This effect is linked to the drug class action of increased glycosuria through the SGLT2 inhibition, which increases the risk of glucose accumulation at the urinary tract level and then the risk of bacteria proliferation [44]. For this reason, this risk could be reduced if patients are adequately instructed in regular personal intimate hygiene [44]. Moreover, it appears that this effect does not carry the risk of pyelonephritis or upper urinary tract infection [45].

Other adverse effects mentioned in the trials, particularly in CANVAS, are the increased risk of bone fracture and lower limb amputation [46]. However, other trials such as DECLARE and EMPAREG, or large population studies, have not confirmed these results, highlighting a risk of incurring such adverse effects similar to that for other antidiabetic drugs [47,48,49,50]. Finally, SGLT2-Is could predispose to dehydration and increased risk for falls; therefore, this class of drugs requires caution when prescribed to the older population [44].

## 3. The Mechanism of Kidney Glucose Reabsorption

### 3.1. In Healthy Subjects

The kidney plays a key role in the balance of glucose metabolism. Under normal circumstances, the kidney is responsible for 20–25% of the endogenously derived glucose through gluconeogenesis, and of filtration and reabsorption from pre-urine of approximately 180 g per day of glucose [51]. The filtered glucose load elevates as glucose plasma concentration increases with full reabsorption until the system reaches saturation, which normally occurs when glycemia is around 180–215 mg/dL. Higher glycemic levels lead to glycosuria, and the maximum renal reabsorption capacity is about 375 mg/min in a healthy individual [52,53].

**Figure 3:**
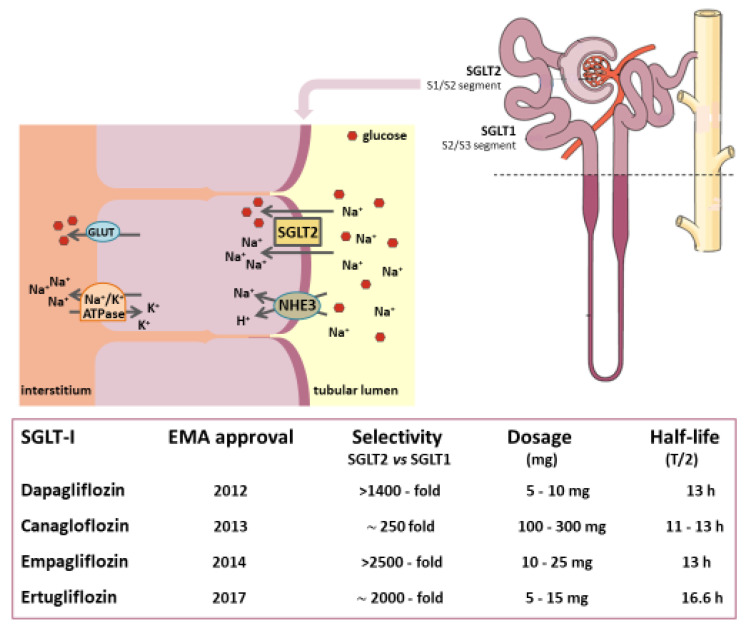
Reabsorption of glucose and sodium in the renal proximal tubule.

The SGLTs are a family of six isoforms of proteins and mediate the transport of glucose, ions, osmolytes, vitamins, and amino acids. Among these, SGLT2 is a symporter located on the apical membrane of the S1 portion of the proximal tubule cells (PCTs). SGLT2 symporters cotransport glucose and sodium in a 1:1 ratio. They show a high affinity and a high capacity to move glucose across the luminal membrane actively, and this active transport occurs against the concentration gradient [54,55]. At the anti-luminal site, glucose leaves the intercellular space by passive diffusion via GLUT1 and 2 transporters, whereas sodium is extruded by an active outward movement driven by ATP [56,57] (Figure 3).

In the kidney of normal subjects, approximately two-thirds of the total sodium reabsorption occurs in the proximal tubule in exchange for H^+^ through the sodium-hydrogen exchanger (NHE) 3. The latter is an isoform primarily expressed in renal and gastrointestinal cells. The NHE3 exchangers colocalize with SGLT2 symporters, and their activities are linked via the accessory membrane-associated protein 17 [58]. As a result, the increased activity of one may increase the activity of the other and vice versa, which is why SGLT2-Is can block NHE3 [59,60]. As proof of this reciprocal influence, NHE3 was upregulated during hyperglycemia in vitro and in the diabetic state in vivo [61]. Interestingly, upregulation of NHE3 via transcriptional, translational, and post-translational mechanisms was also described in the proximal tubule of rats suffering from HF [62]. On the other hand, tubular NHE3 knockout in a mouse model of T1DM decreased SGLT2 expression and inhibited natriuresis induced by SGLT2-Is [63]. Likewise, SGLT2 inhibition was associated with a marked inhibition of NHE3, even in the absence of glucose. This result can explain the significant SGLT2-I-induced natriuresis as demonstrated in proximal tubules of non-diabetic rats perfused with phlorizin [64].

The SGLT1 isoform, primarily found in the gastrointestinal tract and in the S2 and S3 portions of PCT, has a higher affinity but a lower capacity than the SGLT2 isoform. It reabsorbs about 10% of filtered glucose with a glucose to sodium coupling ratio of 1:2 [65]. Blockage of SGLT2 can be partially compensated by SGLT1 activity, which can provide up to 50% of glucose reabsorption as a double uptake of sodium is required for each molecule of glucose absorbed [66].

Work on SGLT1 and SGLT2 knock-out mice allowed a better understanding of the mechanisms and effects of inhibition of these receptors and opened up new therapeutic avenues in humans [67]. These studies showed not only a reduction in plasma blood sugar in the early stages but also a lower loss of β pancreatic cells, thus preserving their pancreatic insulin storage [28,68]. Moreover, it has been shown that such effects appear even in the presence of SGLT1 inhibition alone, particularly for what concerns the stimulus to secrete GLP-1 and the increased glucose tolerance. [69]. A study in which SGLT1 knockout mice were treated with dapagliflozin demonstrated an association with reduced hyperfiltration, blood glucose, kidney weight, glomerular size, and microalbuminuria [70]. These findings may suggest an additive action between SGLT1 and SGLT2 inhibition.

### 3.2. In Diabetic Patients

The capacity of renal glucose reabsorption is enhanced in diabetes due to an SGLT2 overexpression in the PTCs (proximal tubule cells), which can be explained by their persistent exposure to high glucose (HG) levels [71]. Investigations in animal models of diabetes reported an increase in SGLT2 mRNA levels by 38–56% associated with an enhanced expression of the hepatocyte nuclear transcription factor-1α (HNF-1α) [72,73]. A recent study has suggested that plasma glucose levels regulate both expression and function of this transcription factor which specifically would rule the expression of SGLT2 [74]. Consequently, diabetic patients have a higher threshold for urinary glucose excretion and higher glucose reabsorption than healthy humans [75,76]. These pathophysiological changes minimize glycosuria and create a vicious circle that exacerbates glucose accumulation.

It is known that the apical glucose uptake is strictly connected to the basolateral Na^+^/K^+^-ATPase activity. This means that under hyperglycemic conditions, the increase in the sodium-coupled glucose reabsorption via SGLT1 and 2 leads to a reduction in sodium concentration in the downstream tubular lumen [77]. At the end of the Henle loop, this concentration is falsely perceived as an effective hypovolemia by the macula densa of the juxtaglomerular apparatus, and this triggers the TGF. In particular, high sodium levels in the cells inhibit the conversion of ATP into the potent vasoconstrictor adenosine. As a result, fewer adenosine receptors are activated, leading to a reduction in vasodilation of afferent arteriole. In contrast, the intrarenal activation of the renin-angiotensin-aldosterone system (RAAS) constricts the efferent arteriole [78,79]. The resulting increase in intraglomerular pressure induces hyperfiltration and glomerular injury with urinary albumin excretion. An absolute and supraphysiologic elevation in the glomerular filtration rate (GFR) is observed early in the natural history of 10–67% and 6–73% of T1DM and T2DM patients, respectively [80].

To meet the synthesis of ATP required for the transporters to function, the renal oxygen demand is physiologically very high, second only to that of the heart. As approximately 80% of the kidney energy is spent on the reabsorption of sodium from the pre-urine, the majority of oxygen consumption occurs in the PTCs [81]. Therefore, another consequence of glycosuria is the increase in energy expenditure for the Na^+^/K^+^-ATPase activity and the rise in oxygen demand by the renal cortex and the outer medulla determining local hypoxia [82]. This was demonstrated in animal models of diabetes, where renal oxygen consumption increased by 40% in the cortical segments and by 16% in the collecting duct segment [83].

Other mechanisms make the diabetic kidney particularly exposed to hypoxia. Genes regulated by the hypoxia-inducible factor 1α (HIF-1α), including erythropoietin, are not increased in diabetic kidney disease (DKD). This is most probably due to the hypoxia-related tubulointerstitial dysfunction, which results in a decreased number of red blood cells and a further reduction in oxygen delivery [84,85]. In addition, hypoxia stimulates the production of inflammatory and pro-fibrotic pathways determining loss of peritubular capillaries and ischemic injury exacerbating hypoxia [86].

## 4. Effects of SGLT2-Is on CV and Renal Outcomes: Brief Overview of Results from Main Trials

Although the target organs affected in diabetes are many, cardio-renal complications have the greatest impact in terms of morbidity, mortality, and burden on the National Health System [87,88,89,90,91]. Four CVOTs using empagliflozin, canagliflozin, dapagliflozin, and ertugliflozin in T2DM patients have been conducted. Primary outcomes were a 3-point MACE, i.e., a combination of CV mortality, non-fatal myocardial infarction, and non-fatal stroke [46,47,48,92]. The primary outcome of another CVOT assessing sotagliflozin in patients with T2DM, CKD, and risks for CVD changed mid-trial from the 3-point MACE to the total number of CV deaths due to lack of funding [93].

The diabetic population enrolled had a high risk for CV events or an established CV disease. The renal function was variously compromised, but eGFR was at least ≥30 mL/min/1.73 m^2^.

All these trials showed a reduction in CV events in the treated groups compared to placebo, but only empagliflozin and canagliflozin demonstrated a significant effect on the 3-point MACEs.

The main results of EMPAREG OUTCOME, CANVAS, and DECLARE-TIMI 58 were summarized in a meta-analysis performed by Zelniker et al. This analysis showed that improved CV outcomes were achieved in T2DM patients with atherosclerotic CVD, whereas no difference emerged in primary prevention [94]. These findings endorse the 2020 European Society of Cardiology guidelines recommendations for SGLT2-I administration as first-line therapy in patients with T2DM and established CVD [95].

In addition to the primary outcome, several secondary or exploratory endpoints were collected from these trials, including those relative to heart failure (HF) and kidney disease.

Surprisingly, all gliflozins significantly reduced ~30% of hospitalization for heart failure, both in new-onset or recurrent HF. Hospitalization for heart failure reduction rather than prevention of atherothrombotic events was, therefore, considered the major benefit associated with the use of gliflozins in CV patients [94]. Moreover, as hospitalization for heart failure reduction was observed after 2–3 months of treatment, it is unlikely that this favorable CV outcome was dependent on other gliflozins-associated effects such as lowering of glycemia and improvement of other atherosclerotic risk factors [96].

Concerning kidney disease, three CVOTs in T2DM patients demonstrated the ability of SGLT2-Is to reduce a composite of renal outcomes by 40–70%, including doubling of serum creatinine, development of macroalbuminuria, need for dialysis and/or transplantation and kidney death [46,47,48]. Notably, albuminuria and GFR have been highly associated with the risk of cardiovascular events in subjects with type 2 diabetes [97,98,99]. Based on these striking results, additional trials were designed to test the SGLT2-I specific effects on HfrEF. DAPA-HF, EMPEROR-Reduced, and others, as SOLOIST-WHF trials have investigated the combined SGLT2 and SGLT1 inhibitor in T2DM patients hospitalized for HF. The DEFINE-HF trial has evaluated the improvement in HF symptoms, and EMPERIAL trials have tested the response to 6-min walk tests. Concerning CKD endpoints, CREDENCE in patients with DKD and DAPA-CKD in patients with any form of CKD, as well as other minor studies such as DELIGHT, DERIVE, and DIAMOND, were designed in populations with or without diabetes, at risk for or suffering from these cardiac or renal pathologies [100,101,102,103,104,105,106,107,108,109].

Both dapagliflozin in the DAPA-HF and empagliflozin in the EMPEROR-Reduced trial achieved the primary composite outcome of reduced worsening HF and death from CV causes. The results were consistent in different subgroups regarding sex, T2DM, renal function, and current HF therapy, including angiotensin receptor-neprilysin inhibitors. In both trials, SGLT2-Is also significantly reduced adverse renal outcomes.

Overall, the trials investigating kidney endpoints indicated that SGLT2-Is reduced the incidence of renal outcomes to more than 40% in patients with T2DM with or without prevalent CVD. It also delayed CKD progression in both diabetic and non-diabetic patients and lowered the incidence of major adverse CV events and other CV outcomes in patients with renal impairment. The highest CV benefit was observed in diabetic subjects with an estimated-GFR <60 mL/min per 1.73 m^2^. The positive results of the CREDENCE trial led in 2019 to FDA approval of canagliflozin for the treatment of DKD [105]. A brilliant result was shown in the most recent DAPA- CKD trial performed in CKD patients with and without T2DM. Dapagliflozin not only significantly improved the prognosis of important renal parameters but also reduced the combined risk of death from CV causes or hospitalization for heart failure by 29%. Furthermore, dapagliflozin reduced all-cause mortality by 31% [106]. Therefore, both CREDENCE and DAPA-CKD represent two milestones in the cardiorenal protection of T2DM patients and in treating CKD with CV prognosis. Moreover, as DAPA-HF, EMPEROR-Reduced and DAPA–CKD clinical trials also included non-diabetic participants in their studies, this suggests that the clinical benefits of this pharmacological treatment also apply to patients without T2DM. The evidence of a diabetes-independent therapeutic effect was stronger in HF than in kidney disease.

Based on these results, the international cardiology societies promptly updated their guidelines on HF treatment. They recommended using either dapagliflozin or empagliflozin in patients with HFrEF who were already on guideline-directed medical therapy [110,111]. This aimed to reduce HF events and CV death in these patients and was independent of the presence of T2DM.

Likewise, the current American Diabetes Association guidelines recommend using an SGLT2-I with demonstrated CV benefit (empagliflozin, canagliflozin) in T2DM patients with either established atherosclerotic CVD or multiple CV risk factors to reduce the risk of major adverse CV events and/or HF hospitalization. This included both patients already treated with metformin and those commencing treatment for diabetes. In T2DM patients with established HFrEF, a SGLT2-I with proven benefit (empagliflozin, canagliflozin, dapagliflozin, ertugliflozin) is recommended to reduce the risk of worsening HF and CV death [112]. The same guidelines recommend treating T2DM patients with DKD (estimated GFR ≥ 25 mL/min/1.73 m^2^ and urinary albumin ≥ 300 mg/g creatinine) with canagliflozin, empagliflozin, or dapagliflozin to reduce renal disease progression and CV events. Moreover, it was also considered the use of SGLT2-Is for CV risk reduction in T2DM patients with non-diabetic CKD (eGFR ≥ 25 mL/min/1.73 m^2^ or urinary albumin creatinine ≥ 300 mg/g) [113].

The management of HF with preserved ejection fraction (HFpEF) was a major unmet clinical need until October 2021, when the results of the EMPEROR-Preserved trial were published. This study showed for the first time that empagliflozin treatment in patients with EF > 40% and NYHA class II–IV determined a 21% risk reduction of the primary composite outcome of CV death or hospitalization for heart failure. This result was mainly related to a 29% lower risk of hospitalization for heart failure rather than any substantial effect on CV death. The benefit was consistent across patients with or without diabetes [114]. The positive effect was registered 18 days after randomization, in both in- and out-patient HF events, with a similar risk reduction for EF of >40% to <50% and 50% to <60%, even if attenuated at higher EF values [115]. Notably, the enrolled patients had a higher burden of comorbidities, more severe cardiac dysfunction, higher median NT-proBNP and greater use of mineralocorticoid receptor antagonists compared with previous HFpEF trials [116]. These results could inaugurate a new era for the management of HFpEF. Updating of guidelines from cardiological and diabetological societies is expected.

The main characteristics of clinical trials evaluating CV and renal outcomes among patients treated with SGLT2-Is are reported in Table 1 and Table 2.

## 5. The Renal Effects of SGLT2 Inhibition: Correction of Hyperfiltration, Albuminuria, and Hypoxia

SGLT2 inhibition corrects the primary PTC hyper-reabsorption typical of diabetic disease. SGLT2-Is enhance glycosuria and natriuresis, recover the TGF by increasing sodium delivery to the macula densa, and promote vasoconstriction of afferent and vasodilation of efferent arterioles [59]. Although further studies are needed, the interaction between NHE3 and SGLT2 provides a potential mechanism via which SGLT2-Is may exert renal protection in diabetes, since the central role of sodium is delivered to the macula densa cells in the resetting of TGF deranged by hyperglycemia [117].

By modulating renal hemodynamics, SGLT2-Is can reduce glomerular hypertension and hyperfiltration followed by barotrauma and albuminuria. These are all early events of great relevance in the prevention of DKD, and thus SGLT2-Is attenuate its progression in the long term. This is observed in rodent models of diabetes but is also well documented in young and otherwise healthy T1DM hyper-filtering patients [118,119,120,121]. In a more recent study in a murine model of T1DM, empagliflozin increased urinary adenosine excretion and reduced hyperfiltration via afferent arteriolar constriction measured in vivo using multi-photon microscopy [122]. The renoprotection demonstrated by large prospective trials in T2DM patients could depend on different mechanisms from those described for T1DM. A randomized controlled trial (RCT) compared the hemodynamic effects of 3-month therapy with gliclazide vs. dapagliflozin in T2DM patients with eGFR > 60 mL/min/1.73 m^2^ and overt proteinuria. The study demonstrated that SGLT2 inhibition reduced measured GFR and filtration fraction without increasing renal vascular resistance. This suggests a possible role for post-glomerular vasodilation, possibly mediated by an increased production of prostaglandin preventing TGF-mediated pre-glomerular vasoconstriction [123]. The arterial stiffness typical of older T2DM patients could limit the potential for preglomerular vasoconstriction, thus explaining the lower intraglomerular pressure and less prominent hyperfiltration compared to T1DM patients.

As reported in several studies, the renal hemodynamic changes by SGLT2-Is may result in an initial drop of eGFR followed by stabilization, the so-called “checkmark” sign (√) [122,124,125,126]. Long-term treatment with SGLT2-Is compared to placebo is associated with a slower decline of GFR and a reduction of albuminuria by 30–50% in treated T2DM patients [127,128]. These endpoints were achieved independently of blood pressure (BP) changes or glucose control, even in subjects with overt DKD [129,130].

The increased albumin excretion observed in both T1DM and T2DM patients with DKD appears mainly related to alterations in the glomerular filtration barrier and podocytes [131,132]. In diabetic and non-diabetic mice with kidney injury induced by bovine serum albumin injection for three weeks, dapagliflozin limited proteinuria, damage and dysfunction of glomeruli, and loss of podocytes to a similar extent as an ACE inhibitor [133]. Interestingly, the study identified the presence of SGLT2 in podocytes and increased mRNA and protein expression after induction of proteinuria, thus indicating another potential mechanism of SGLT2-I nephroprotection.

Chronic hypoxia is apparent in the development and progression of CKD, including DKD. Therefore, the increased renal oxygen tension, secondary to reduced oxygen utilization for sodium reabsorption, may contribute to the nephroprotective effects of SGLT2-Is [134,135].

Based on the demonstration of the HG-enhanced activity and expression of NHE3 in mesangial cells, it can be hypothesized that NHE3 inhibition in these cells represents a potential mechanism by which SGLT2-Is could prevent glomerulosclerosis and ameliorate DKD [136].

## 6. Direct Effect of SGLT2-Is on Myocardial Sodium Homeostasis

Despite the negligible expression of SGLT2 in the heart, SGLT2 inhibition is strongly implicated in cardiac sodium homeostasis because of its profound effects on ion transporters [137].

The sodium content of cardiomyocytes plays a critical role in cardiac electro-mechanical coupling and contractility and the mitochondrial processes of oxidation-reduction. Therefore, it is unsurprising that dysregulated Na^+^ handling is centrally involved in the development and progression of HF [138].

Na^+^ homeostasis in cardiomyocytes is tightly coupled with that of Ca^2+^ through the activity of multiple ion channels, including sarcolemmal Na^+^/Ca^2+^ exchanger (NCX), which exchanges 3 Na^+^ for 1 Ca^2+^ and mitochondrial Na^+^/Ca^2+^ exchanger (NCLX). Their activity is closely related to calcium concentration. Physiologically, NCX mainly removes calcium from the intra- to the extracellular space, except in the early phase of action potential when, independent of the [Na^+^] and [Ca^2+^] transmembrane gradient, NCX transports Ca^2+^ in the reverse direction into the cytosol. NCLX is mainly responsible for mitochondrial Ca^2+^ release into the cytosol [139].

In HF, the concentration of Na^+^ in cardiomyocyte cytosol is significantly increased due to an imbalance between ion influx and efflux. Cytosolic Ca^2+^ concentration is also elevated secondary to increased efflux from the mitochondria via NCLX [140]. Lowering intra-mitochondrial Ca^2+^ concentration suppresses the Ca^2+^-dependent upregulation of dehydrogenases in the tricarboxylic acid cycle to increase the production of reducing equivalents. The result is a decrease of NADH with less ATP production and decreased NADPH with disruption of mitochondrial antioxidant cellular defenses. Both Na^+^ and Ca^2+^ cytosolic overload activate Ca^2+^/Calmodulin-dependent kinase II (CaMKII), a serine/threonine kinase with a central role in regulating multiple Na^+^ and Ca^2+^ channels/transporters. Ca^2+^/CMKII is markedly upregulated in HF and is implicated in arrhythmogenesis and adverse myocardial remodeling [141,142].

Overall, the dysregulation of Na^+^ and Ca^2+^ homeostasis typically present in HF contributes to systolic, diastolic, and mitochondrial dysfunction, and to arrhythmogenesis and detrimental remodeling [143]. The raised concentration of Na^+^ in a failing myocardium has mainly been associated with three factors, the enhancement of the late sodium current (I_NaL_) responsible for significant Na^+^ influx, the hyperactivity and hyperexpression of sarcolemmal NHE1, the major NHE isoform present in the heart, which couples the extrusion of one H^+^ with the influx of one Na^+^, and the upregulation of NCX expression and activity [144,145,146,147]. Hyperexpression of SGLT1 transporter mRNA or protein could also contribute to sodium overload, whose presence has repeatedly been confirmed in healthy hearts instead of SGLT2. Reduced capacity of Na^+^ extrusion from the cytosol, a debated process mediated by the Na^+^/K^+^ ATPase, may also contribute [139,148].

As found in preclinical studies, the aforementioned alterations may be in part corrected by SGLT2-Is. The inhibition of NHE1 in an elevated glucose environment by empagliflozin acutely decreased cytoplasmic Na^+^ and Ca^2+^ concentrations, increased mitochondrial Ca^2+^, and improved myocardial contractility in rats and rabbits [149]. In in vitro studies, gliflozins reduced the sodium concentration within mouse and human cardiomyocytes, possibly by directly inhibiting NHE-1 [150,151]. An inhibitory effect of SGLT2-Is on cardiac I_NaL_ was recently demonstrated in a mouse model of HF [152].

SGLT2-Is could directly inhibit two members of the SGLT family, SGLT1 and the sodium–myoinositol cotransporter-1 (SMIT1), both of which are hyper-expressed and hyperactive in the cardiomyocytes of diabetic patients. Except for sotagliflozin, which was designed as a dual SGLT1/SGLT2 inhibitor, the other gliflozins exert high selectivity for SGLT2 over SGLT1, making a significant contribution to SGLT1 inhibition in vivo unlikely but worthy of further investigation (Figure 3) [153].

Empagliflozin has been shown to counteract abnormal Ca^2+^ handling in HF by reducing CaMKII activity [154]. The reduction of Na^+^ overload in HF with SGLT2-I treatment decreases reactive oxygen species (ROS) production and the occurrence of oxidative stress [155]. The result of the above mechanisms leads to improved systolic and diastolic function with reduced risk of arrhythmia.

## 7. Improvement of Conventional CV and Renal Risk Factors by SGLT2-Is

Previous research on anti-hyperglycemic or multifactorial treatment in diabetes found that even if a reduction in CV events was achieved, this benefit only becomes apparent after years of treatment [156]. Instead, the mortality benefit attributed to SGLT2-Is appeared within a short timeframe, as indicated by the early separation of the Kaplan–Meier curves in the CVOTs [157,158]. Given this rapid occurrence, the outcomes are unlikely explained by the improvement of conventional risk factors. Nevertheless, a role cannot be completely excluded since even direct beneficial actions on the heart and kidneys can be affected by a systemic setting influenced by the amelioration of these parameters.

### 7.1. Improved Glucose Control

The CV benefits of SGLT2-Is are unlikely due to the correction of hyperglycemia-related metabolic abnormalities for three simple reasons. They are disproportionately great for the relatively small reduction of HbA1c (0.6–1.0%), they exert a similar effect in diabetic and non-diabetic patients, and the pursuit of an intensive glycemic control in large clinical trials designed ad hoc did not translate into a significant CV outcome benefit. Moreover, a mediation analysis of EMPA-REG OUTCOME by Inzucchi et al. showed a slight contribution of HbA1c reduction to the observed cardioprotective effects of empagliflozin [159]. As well as hypertension, chronic hyperglycemia is a major risk factor for developing DKD, a progressive disorder that represents the worldwide leading cause of both dialysis and transplantation [160].

In the kidney, cellular glucose uptake is non-insulin-dependent and increases in proportion to the plasma concentration. In a hyperglycemic context such as diabetes, glucose overloads, glomerular cells, and PTCs are diverted into non-glycolytic pathways. This results in glycosylation of proteins, generation of advanced glycation end (AGE) products, and activation of the corresponding receptors (RAGEs), which promote mitochondrial dysfunction, oxidative stress, inflammation, and apoptosis [161]. By reducing plasma glucose levels, SGLT2-Is, like any glucose-lowering strategy, may prevent these adverse metabolic effects. Nevertheless, the renoprotection observed in the CREDENCE trial is out of proportion concerning the modest reduction of blood glucose levels, and drugs with greater glucose-lowering effectiveness did not produce the same rapid and marked benefits as SGLT2-Is [105].

### 7.2. Loss of Body Weight

SGLT2-Is have been specifically designed to promote glycosuria and thus loss of calories, a unique feature compared to all the other glucose-lowering drugs. A daily energy waste of approximately 200–300 kcal has been calculated; therefore, weight loss is expected, contributed to by the decreased insulin secretion and activation of catabolic pathways such as increased lipolysis and fat oxidation, as described later [162].

In rats with high-fat diet-induced obesity, dapagliflozin demonstrated greater efficacy in improving peripheral insulin sensitivity and reducing weight gain than vildagliptin [163]. Similar results were obtained in obese mice with streptozotocin-induced diabetes treated with dapagliflozin and liraglutide [164]. A clinical trial on the use of SGLT2-Is registered a weight loss of approximately 2 to 3 kg during the initial 6–12 months of therapy. To further confirm the weight loss associated with the use of this class of drugs, a meta-analysis on 29 RCTs with a low risk of bias showed that in a total of 12,000 patients, the mean weight loss ranged from −2.26 kg to −0.79 kg when patients were treated with 300 mg canagliflozin and 25 mg ipragliflozin, respectively [165]. Initially, the weight loss is mainly due to fluid depletion, but subsequent fat tissue is lost, including that from visceral depots [166]. In the real world, the impact of SGLT2 inhibition on body weight seems to be significantly lower than anticipated in the clinical trials studies [167]. Although an increased body mass index is a well-known CV risk factor, independently associated with a greater risk for CKD and ESRD, the modest weight loss cannot explain the significant reduction in CV mortality. This may also partially contribute to kidney protection exerted by SGLT2 inhibition [168,169,170].

### 7.3. Reduction of Arterial Blood Pressure

SGLT2 inhibition has been shown to increase by 67% the survival of spontaneously hypertensive rats and to change the BP circadian rhythm from a non-dipper to a dipper profile in animals and human studies [171,172]. A meta-analysis of various clinical trials documented a BP decline of comparable entity to low-dose hydrochlorothiazide. The BP decline was quantified as –3.62 mmHg for systolic and −1.70 mmHg for diastolic BP, regardless of the SGLT2-I dosage [173]. BP lowering is likely dependent on a combination of osmotic diuresis and mild natriuresis due to a higher tubular lumen glucose concentration and urine sodium excretion, respectively [174]. It has been calculated that these effects induce a negative water and sodium balance of ~700 mL and ~100 mEq within the first 48–72 h of therapy [59]. As intravascular volume is mildly depleted, this may suggest that SGLT2-I therapy activates RAAS. The effects of RAAS activation, however, do not include a reduction in angiotensin levels, which can be attributed to the anti-hypertensive effect. The association between RAAS and AGLT2-inhibition is simple. Plasma renin activity increases in the first three to six months of treatment, but remains unchanged in the long-term therapy similar to aldosterone [175].

Several anti- contributor mechanisms have been proposed that could explain the anti-hypertensive effect of SGLT1-is. Body weight reduction is one, and accounts for about 40% of the change in BP. Other mechanisms are the controversial suppression of the sympathetic nerve activity and the improvement in endothelial dysfunction and arterial stiffness [176,177,178,179,180]. Nevertheless, it is difficult to account for the modest antihypertensive effect of SGLT2-Is playing a significant role in improving CV risk. This latter is expected to be achieved after many years and translate into reduced atherothrombosis, with a primary effect on the incidence of strokes rather than cardiac events alone. On the contrary, results from meta-analyses on T2DM patients receiving gliflozin indicated no relationship between BP and CV outcomes and no changes in reducing in risk of stroke [181,182,183].

SGLT2-Is have been demonstrated to be very effective in the prevention of DKD, even more than ACE inhibitors or ARBs, and to enhance RAAS blockage [59]. However, even in the case of renoprotection, the modest drop in BP associated with SGLT2 inhibition suggests that other factors are predominantly involved [184]. Moreover, the intensive control of BP does not seem to prevent end-stage kidney disease in T2DM patients [185].

### 7.4. Reduction of Serum Uric Acid

Uric acid has been associated with CV diseases and with inflammation and oxidative stress. In addition, the majority of these studies showed that elevated levels of uric acid are a potential contributor risk factor in the development and progression of CKD [186,187].

A meta-analysis on 62 RCTs provides relatively robust evidence for a moderate reduction in serum uric acid following SGLT2-Is, which persists during long-term treatment, but is absent in patients with eGFR < 60 mL/min [188]. The effect is likely determined by a uricosuric action possibly associated with increased glycosuria. This was demonstrated in a recent study reporting a suppression of urate reabsorption through glucose transporter 9 and urate transporter in the distal segments of renal tubular cells [189]. Also, in this case, a reduction in the uric acid levels alone is not sufficient to explain the overall clinical benefits of SGLT-Is in cardiorenal protection. It is more likely that, instead, this is just one of the several therapeutic advantages.

### 7.5. Effects on Serum Lipids

Even if a decrease of low-density lipoprotein cholesterol (LDL-C) levels is described in a retrospective study in diabetic patients newly treated with dapagliflozin, a slight but net increase in LDL-C levels is mostly observed in clinical studies with SGLT2-Is [190,191,192,193]. Interestingly, this adverse effect not consistent with the cardiorenal benefit of gliflozins seems to be counteracted by favorable changes in the ratio of both LDL and HDL subclass particles. Hayashi et al. showed that a 12-week dapagliflozin therapy in T2DM patients decreased by 20% the potent atherogenic small dense LDL-C, and increased by 18% the large buoyant less atherogenic LDL-C [194]. Concomitantly, an 18% increase of the favorable high-density lipoprotein cholesterol 2 (HDL2-C) without modifications of HDL3-C was documented. In the same study, no effect on plasma lipids and lipoprotein subspecies was associated with sitagliptin administration. In a small multicenter prospective study in 22 T2DM patients, 12 weeks of canagliflozin taking increased the very large and large HDL lipoproteins by 10.9% and 11.5%, respectively [195].

Moreover, a reduction of triglyceride levels is described in both clinical and preclinical studies with SGLT2-Is [190,196].

The mild decrease of triglyceride and increase of both HDL-C and LDL-C levels are confirmed by a recent meta-analysis including 36 RCTs in T2DM patients using a gliflozin for at least 24 weeks [197].

From a pathophysiological point of view, SGLT2-Is might increase the liver provision of cholesterol substrates through enhancement of lipolysis, leading to increased cholesterol synthesis, decreased activity of LDL receptors, and finally, increased serum LDL-C level. On the other hand, the increased lipolysis and the improved insulin sensitivity determined by attenuation of systemic glucotoxicity might reduce the serum levels of triglycerides and thereby increase HDL-C [198].

Two observations may arise from these findings. On the one hand, the changes in lipid profile induced by SGLT2-Is fit perfectly with the correction of the highly atherogenic diabetic dyslipidemia, a triad notoriously characterized by an increase of triglycerides and small dense LDL-C and a decrease of HDL-C [198]. On the other hand, these minor lipidemic modifications may only represent ancillary players concerning the wide range of beneficial pleiotropic mechanisms behind the remarkable cardiorenal benefits of SGLT2 inhibition [199].

### 7.6. SGLT2-Is and Atherosclerosis

The positive impact of SGLT2-Is on the development and progression of atherosclerosis cannot be disregarded regarding the data reported above.

On the other hand, clinical data collected so far cannot prove a delay in the progression of atherosclerosis. Despite the significant reduction in CV mortality, CVOTs with SGLT2-Is demonstrated no benefit in reducing non-fatal atherosclerotic CV events, such as myocardial infarction and stroke. This finding is possibly due to the prevalent role of other mechanisms, and perhaps to the short observation time [46,47,48]. The issue also remains open regarding the many human and animal data suggesting multiple possible pathways mediated by SGLT2 inhibition, not only through glucose-lowering, but also lipid metabolism and foam cell formation, vascular inflammation and oxidative stress, endothelial dysfunction, and prevention of platelet activation, as discussed in recent reviews [200,201].

## 8. Metabolic Reprogramming by SGLT2-Is

In patients treated with SGLT2-Is, the acute reduction in plasma glucose levels, secondary to glycosuria, leads to a lower plasma insulin concentration and insulin-to-glucagon ratio. A fasting-like state of energy depletion is established when glucose entry into cells is reduced, lipolysis is intensified, and ketogenesis stimulated. This results in elevated circulating fatty acids (FAs) and ketone bodies, mainly β-hydroxybutyrate (β-HB) [18,202]. In these conditions, both heart and kidney shift from glucose to FA and ketone body utilization [203,204].

In a model of obese non-diabetic mice, canagliflozin led to a loss of BW and improved glucose tolerance, although plasma insulin is decreased. In addition, canagliflozin increased plasma ketones and improved plasma lipid profiles [205]. The study demonstrated that this reduction in fat mass and stimulation of lipolysis is mediated by the activation of the fibroblast growth factor 21, a powerful but still debated hormone involved in the modulation of energy homeostasis and metabolism [206]. In individuals with T2DM, dapagliflozin ameliorated insulin sensitivity and caused a shift from glucose to lipid oxidation, which, together with the increased glucagon-to-insulin ratio, are the pathophysiological bases for the enhanced ketone synthesis [202].

Even if the SGLT2-I-induced metabolic reprogramming is well documented, its relevance and significance for cardiorenal protection are still controversial. Some researchers have recently hypothesized that SGLT2-I controls cell life programming and promotes a switch from a defense to an energy-saving dormancy state [207].

### 8.1. Metabolic Effects of SGLT2 Inhibition at Heart Level

Under normal conditions, FA metabolism and carbohydrate oxidation contribute 60% and 40%, respectively, to cardiomyocytes’ energy supply. Under stress, carbohydrates become the main fuel for the heart as they can instead be metabolized by glycolysis under anaerobic conditions, even though this leads to a much lower ATP production compared to that generated during mitochondrial oxidation. On the other hand, FAs oxidation requires a greater oxygen consumption than that used by glucose as the ATP production/O2 consumption (P/O) ratio is 2.33 for FAs vs. 2.53 for glucose, which could exacerbate an eventual myocardial ischemia [140]. The heart is, by mass, the organ with the highest consumption of ketone bodies, which are a super fuel and can be used as an additional substrate for energy production in HF. The cardiomyocytes of a failing heart are often subjected to an insufficient oxygen supply over long periods. During this lack of oxygen, ketone bodies can be better substrates than FAs at producing ATP due to a more favorable P/O ratio (2.50). Ketone bodies are also better than glucose, even though their P/O values are not significantly different [155,208,209]. β-HB must be converted into acetoacetate to generate energy. The advantage is that before being oxidized, acetoacetate is first activated to acetoacetyl-CoA by succinyl-CoA:3-ketoacid CoA transferase (SCOT), a reaction that does not require ATP. In contrast, the activation of glucose by hexokinase and of FAs by acyl-CoA synthetases requires ATP [210]. Accordingly, HF is characterized by increased transcription of genes associated with the oxidation of ketone bodies. Increased transcription of the gene encoding SCOT is one example; this gene encodes the rate-limiting ketolytic enzyme [211].

HF has also been associated with altered metabolic regulation of branched-chain amino acids (BCAAs), which could be restored by empagliflozin [212,213].

The shift in fuel energetics results in improved myocardial energy efficiency and possibly improved cardiac function [214,215,216]. In a small trial of HF patients, β-HB infusion improved cardiac output by 40% and left ventricle (LV) EF by 8% compared with placebo infusion. Equivalent hemodynamic effects were observed in age-matched volunteers [217]. In a mouse model of prediabetes, empagliflozin treatment improved LV contractility and cardiac microcirculation in combination with an increase in plasma ketone bodies, glucagon concentration, and glucagon/insulin ratio [218]. In a porcine model of ischemic non-diabetic HF induced by left anterior descending artery occlusion, empagliflozin increased the utilization of BCAAs, FAs, and ketone bodies with concurrent improvement in LV function and mitigation of HF remodeling [219]. In a doxorubicin-induced non-diabetic HF mouse model, empagliflozin significantly increased β-HB plasma concentrations and ATP production and prevented the development of cardiomyopathy [220].

In addition to their involvement in energy metabolism, other protective effects have been proposed for ketone bodies, such as a significant reduction of oxidative stress and inflammation and an extended lifespan described in mouse studies [221,222]. The mechanism responsible may be inhibition of class I histone deacetylases by β-HB. This is correlated with enhanced transcription of genes encoding oxidative stress resistance factors (FOXO3A and MT2) and of increased activity of the nucleotide-binding domain-like receptor protein 3 (NLRP3) inflammasomes [221,222].

When administered to T2DM patients, SGLT2-Is induced a greater reduction in IL-1β secretion, increased β-HB, and decreased serum insulin levels than sulfonylureas. In the same study, ex vivo experiments on macrophages demonstrated an inhibitory effect of high β-HB and low insulin concentrations on the activation of the NLRP3 inflammasome [223].

### 8.2. Metabolic Effects of SGLT2 Inhibition on the Kidney

In conditions characterized by elevated levels of circulating FAs and damaged glomeruli such as T2DM and metabolic syndrome, albumin-bound FAs can be filtered, causing abnormal FA reabsorption by the proximal tubule with consequent tubulointerstitial inflammation and renal dysfunction. Upregulation of kidney injury molecule-1 is responsible for this FA-induced pathological process [224]. SGLT2-Is may reduce kidney injury by causing a shift from whole-body glucose to FA oxidation, decreasing the accumulation of toxic lipid metabolites (fatty acyl CoAs, diacylglycerol, and ceramides) in podocytes, mesangial cells, and PTCs. This attenuates oxidative stress, endoplasmic reticulum stress, and pro- inflammatory and fibrotic processes [59].

The relationship between ketone bodies and the kidney is more intricate than for the heart and needs further clarification. Ketones are freely taken up and oxidized by the kidney in proportion to their plasma concentration. They could represent a more efficient energy source considering that a complete switch from FA to ketone oxidation theoretically improves renal oxygen efficiency by 9–10% [59,225]. However, SCOT is expressed at lower levels in the kidney than myocardium, indicating comparatively lower use of ketone bodies as an energy source [215,226]. If so, the accumulation of non-utilized ketones could promote beneficial effects against inflammation, oxidative stress, and fibrosis, as described in the heart [210]. On the other hand, past experiments demonstrated that plasma ketone concentrations dilate the afferent arterioles, thereby promoting glomerular hyperfiltration that may exacerbate diabetic nephropathy [227]. More recent studies reported that the diabetic kidney is characterized by enhanced ketogenic activity, even in the absence of SGLT2 inhibition, because of increased expression of the key enzyme 3-hydroxy-3-methylglutaryl-CoA synthase 2. Excess ketones may be involved in the pathogenesis of DKD [228].

## 9. Improved Heart Overload by SGLT2-Is: Effect on Diuresis and Vascular Function

Despite the lack of SGLT2 expression in the heart, SGLT2-Is may reduce HF events within a short period. This effect could be associated with improved ventricular loading conditions due to early hemodynamic effects. This involves the simultaneous reduction in preload, secondary to natriuresis and osmotic diuresis, and in afterload, through lowering BP and correction of vascular dysfunction [229].

### 9.1. Impact of the Diuretic Effect of SGLT2-Is on Intra- and Extravascular Volumes

As described in animal models treated with dapagliflozin and in various human studies with dapagliflozin, canagliflozin, and empagliflozin, SGLT2-Is may modestly reduce plasma volume by inducing natriuresis and osmotic diuresis [230,231,232,233]. In a study of patients with T2DM, a condition characterized by sodium retention, SGLT2 inhibition with dapagliflozin significantly decreased the skin sodium content after 6 weeks [234]. A mediation analysis of the EMPA-REG OUTCOME trial confirmed the importance of plasma volume contraction, indicating that hemoconcentration accounted for approximately 50% of the observed CV benefit [159].

On the other hand, the importance of correcting plasma volume overload in CV outcomes by SGLT2-Is may be questionable because of multiple observations. In a secondary analysis of the EMPEROR-Reduced trial, the magnitude of benefit was not significantly different in patients with recent volume overload [235]. In the DAPA-HF trial, treatment with dapagliflozin did not necessitate a reduction of diuretic dosage [100]. The change in plasma volume seems to be a transient early occurrence that is not sustained in the long term [232,233,236]. For example, an open-label study of 22 patients receiving empagliflozin for 5 days reported that sodium excretion and urine volume increased after the first dose but returned to baseline after multiple doses [237]. It is likely that the kidneys rapidly adapt via compensatory sodium reabsorption at more distal tubular segments to maintain a neutral sodium balance. Finally, loop diuretics have not changed HF prognosis despite being used for decades to increase sodium and fluid excretion, likely because of concomitant detrimental neurohormonal changes [238,239].

The effects of diuretics differ in several respects from those of SGLT2-Is; the main difference is that diuretics directly antagonize sodium entry into the macula densa leading to increased RAAS and systemic sympathetic activity [233]. Instead, the proximal tubular location of the SGLT2-Is action leads to increased sodium chloride delivery to the macula densa, which could account for the small or absent activation of neurohormonal and sodium retaining response compared to what would normally be expected in response to the observed reduction in blood volume and pressure [240]. This would explain why, in a comparative study of dapagliflozin and hydrochlorothiazide, a reduction in plasma volume and an increase in erythrocyte mass was observed with dapagliflozin but not with hydrochlorothiazide over a 12-week treatment period [231].

Other data indicate that the diuresis induced by SGLT2 inhibition is expected to produce a greater electrolyte-free water clearance compared with classical Na+-driven diuretics. The hypothesis of differential regulation of intra- and extravascular volumes by SGLT2-Is is suggested by a study in healthy volunteers that described by using a mathematical model, a 2-fold greater reduction in electrolyte free water and thus interstitial fluid after 7 days of dapagliflozin treatment compared to the loop diuretic bumetanide which reduced intravascular volume [241]. In a sub-study of the Empire HF trial, a reduction in the estimated extracellular fluid and plasma volumes was shown in a population of mostly non-diabetic patients with HFrEF treated with empagliflozin [242].

A differential effect of regulating interstitial fluid may be relevant in patients with HF in whom, in many instances, intravascular contraction is present and often aggravated by diuresis. Instead, the contraction of extravascular volume by SGLT2-Is may reduce congestion and pulmonary edema in HF and improve cardiac function via lowering preload [229]. Accordingly, the end diastolic volume observed with cardiac magnetic resonance was reduced in T2DM patients treated with empagliflozin [243].

### 9.2. Improvement of Arterial Function and Stiffness by SGLT2-Is

Another mechanism by which SGLT2-Is could improve cardiac function is a reduction in afterload through the dampening of arterial stiffening, as reported by multiple clinical and preclinical data in diabetes.

In a post-hoc analysis of data from five phase III trials in T2DM patients, empagliflozin was shown to reduce pulse pressure, a surrogate measure of arterial stiffness and vascular resistance [180]. Reduced arterial stiffness by empagliflozin has also been reported in a sub-analysis of a clinical trial of T2DM patients and a study of young T1DM patients [244,245]. A significant increase in shear stress, the main hemodynamic factor stimulating endothelium to produce vasodilators and atheroprotective substances, and in flow-mediated dilation of the brachial artery was observed when comparing treatment with empagliflozin versus incretin drugs. Both treatments resulted in a similar level of glycemic control [246]. The acute treatment of 16 T2DM patients with dapagliflozin significantly improved systemic endothelial function and arterial stiffness independent of BP changes, a finding which was not confirmed in a larger follow-up trial by the same investigation group [247,248]. Canagliflozin and tofogliflozin yielded similar vascular benefits in other clinical studies of T2DM patients [249,250]. Animal studies have confirmed a significant reduction of aortic stiffness and vascular dysfunction by SGLT2-Is. A mechanistic investigation of aortic preparations from animals demonstrated that dapagliflozin induced vasodilatation through activation of voltage-gated potassium (Kv) channels and protein kinase G (PKG) [251,252,253]. The changes in central hemodynamics and aortic function induced by SGLT2 inhibition may be more pronounced than those suggested by the rather small reduction of BP as measured at the brachial artery. RCT evidence has shown that, in type 2 diabetics, empagliflozin reduces central and 24 h systolic and diastolic BP, central pulse pressure, and various vascular parameters of aortic stiffness after 6 weeks of treatment [254]. Overall, the literature supports the argument that SGLT2-Is reduce HF events, at least in part, via improved vascular function, considering that central systolic blood pressure is primarily determined by arterial stiffness of large arteries and an important surrogate parameter afterload strongly linked to future CV outcomes [255]. The mechanisms underlying the amelioration of arterial dysfunction and stiffness by SGLT2-Is in diabetes are not fully understood. Considering the expression of SGLT2 in the arterial wall, modulation of vascular homeostasis via regulation of both endothelial and smooth muscle function and thus arterial remodeling appears likely [256].

## 10. Anti-Inflammatory and Anti-Oxidant Effects of SGLT2-Is

Evaluation of inflammation and oxidative stress, two key pathways known to cause organ damage in diabetes, was not among the aims of the main CV and renal outcome trials of SGLT2-Is. However, a meta-analysis of 15 randomized and 8 observational clinical studies of these drugs showed a consistent reduction in biomarkers of both inflammation (CRP, IL-6, and TNF-α) and oxidative stress (8-iso-prostaglandin F2α and 8-hydroxy-2′-deoxyguanosine) and an increase of the cardioprotective adiponectin derived from adipose tissue [257]. In a recent real-world observational pilot trial, treatment with various SGLT2-Is modified the redox status and antioxidant enzyme activity in the urine of T2DM patients [258].

In addition to the amelioration of hyperglycemia and AGE/RAGE signaling related to glucotoxicity, the correction of multiple risk factors may indirectly confer inflammation-relieving properties to SGLT2-Is.

Gliflozins reduce the adipose tissue excess, a condition closely associated with low-level chronic inflammation characterized by abnormal cytokine and chemokine production and activation of inflammatory pathways that interfere with insulin signaling, including mitogen-activated protein kinases (MAPK), IκB-kinase β (IκKβ)/nuclear factor κB (NF-κB) and mammalian target of rapamycin (mTOR)/S6 kinase [259]. In T2DM patients, canagliflozin decreased circulant leptin by 25% and increased adiponectin by 17% compared with glimepiride [260]. In murine models of obesity, empagliflozin treatment was associated with modulation of NLRP3 inflammasome signaling and activation of fat browning and macrophage polarization to the anti-inflammatory M2 phenotype [261,262]. The reduced mass and inflammation of perivisceral adipose tissue induced by SGLT2-Is may minimize the inflammatory paracrine actions on visceral organs such as the heart and kidney to promote cardiac fibrosis and renal glomerulosclerosis [263]. In particular, SGLT2-Is exert a drug class effect of reduction of epicardial adipose tissue, the true visceral fat of heart, associated with attenuated inflammation and less secretion of deleterious adipokines, that can positively affect both structure and function of adjacent myocardium [264,265]. A particularly relevant role seems to be played by attenuation of leptin secretion and related paracrine effects [266,267].

SGLT2-I treatment results in a slight diminution of circulating uric acid, a waste product released from ischemic tissues and dying cells, notoriously able to activate the NLRP3 inflammasome when crystallized [188,268]. A study on macrophages stimulated with uric acid demonstrated that even the soluble form activates this pathway [269].

SGLT2-I therapy may take advantage of the potent anti-inflammatory properties of ketone bodies that, like lowering insulin and uric acid, reduce the IL-1β secretion by immune cells [270]. In a pilot trial of T2DM patients at high CV risk, despite a similar glucose-lowering effect, empagliflozin determined a greater reduction of IL-1β secretion than glimepiride. This response was associated with increased serum β-HB and decreased circulant insulin, two conditions inhibiting NLRP3 inflammasome activation as verified in the same study in ex vivo macrophages [223].

Strictly connected to the anti-inflammatory property, SGLT2-Is also correct oxidative stress and endothelial dysfunction [271,272]. In diabetic rodents, ipragliflozin mitigated the biomarkers of oxidative stress and inflammation and prevented the development of endothelial dysfunction [273,274,275]. The same responses were evocated by empagliflozin in rat models of T1DM and T2DM [276,277].

Instead, findings on endothelial function from clinical studies are conflicting. In the DEFENCE trial of 80 patients with early-onset T2DM, dapagliflozin improved endothelial functions assessed by flow-mediated dilation and oxidative stress evaluated through the urine biomarker 8-hydroxy-2′-deoxyguanosin [278]. By contrast, in the EMBLEM trial of 117 patients with T2DM and established CVD, a 24-week treatment with empagliflozin did not improve endothelial dysfunction measured with the reactive hyperemia peripheral arterial tonometry index [279].

Experiments in vitro with empagliflozin, therefore not influenced by changes in systemic milieu, suggest that SGLT2 inhibition may directly attenuate inflammation and oxidative stress, determining reduced superoxide production and enhanced expression of glutathione s-reductase and catalase in leukocytes in diabetic patients and downregulating IKK/NF-κB, MKK7/JNK, and JAK2/STAT1 signaling pathways in LPS-stimulated macrophages [280,281].

### 10.1. Evidence of Beneficial Anti-Inflammatory, Anti-Oxidant, and Anti-Fibrotic Effects on Heart

Inflammation is a critical factor in the development and progression of HF and diabetic cardiomyopathy; therefore, a therapeutic target is possibly centered by SGLT2-Is [282].

In diabetic mice, dapagliflozin was proven to halt cardiac inflammation by inhibiting NLRP3 inflammasome, resulting in attenuation of fibrosis and amelioration of LVEF. In the same study, experiments in cardio myofibroblasts in vitro replicated the anti-inflammatory and anti-fibrotic effects, indicating their independency from inhibition of SGLT2 and systemic variations [283]. In HF rodent models, empagliflozin inhibited NLRP3 inflammasome in a Ca^2+^-dependent manner [284]. Other investigations indicated that the activation of AMP-activated protein kinase (AMPK) could be the mediator of myocardium anti-inflammatory effect of SGLT2-Is inhibition [285,286].

Several data document that SGLT2-Is reduce myocardial oxidative stress, a hallmark of cardiac diseases, including HF. An already discussed mechanism is the correction of myocardiocyte Na+ overload that generates the extrusion of Ca^2+^ from mitochondria, impairment of Ca^2+^-induced stimulation of Krebs cycle dehydrogenases, and ultimately deficit of reducing equivalents NADH and NADPH. In turn, the increased formation of ROS can cause cellular Na+ accumulation (e.g., by stimulation of INaL), leading to a vicious cycle that may be stopped by gliflozin [287]. Empagliflozin normalized some oxidative stress parameters (high malondialdehyde, lower antioxidant enzymes GSH-Px, and SOD) and reduced the hyperexpression of NOX4 in hearts of T2DM mouse models. These effects were mediated by translocation of nuclear factor erythroid 2 (Nrf2) into the nucleus and activation of Nrf2/antioxidant response element signaling, the primary pathway involved in regulating oxidative stress [288]. In heart and cardiomyocyte models of isoprenaline-induced cardiac oxidative stress, canagliflozin augmented antioxidant and anti-inflammatory signaling (involving AMPK, Akt, eNOS, Nrf2, and heme oxygenase-1) and attenuated pro-oxidant, pro-inflammatory, and pro-apoptotic responses (mediated by iNOS, TGF-β, Nox4, caspase-3, and Bax) [289].

Since inflammation and oxidative stress stimulate myocardial fibrosis, SGLT2 may favorably affect this process that underlies the structural LV remodeling in diabetic and HF patients [209]. In post-infarcted rats, dapagliflozin attenuated myofibroblast infiltration and cardiac fibrosis via modulation of macrophage phenotype through reactive oxygen and nitrogen species/STAT3-dependent pathway [290]. Empagliflozin reduced cardiac fibrosis in animal models of diabetes and hypertension [288,291]. In more detail, empagliflozin significantly reduced the high level of TGF-β1 expression and Smad1, Smad2, Smad3 proteins involved in the fibrosis pathway, as well as the high levels of type I and III collagen in the myocardium of diabetic mice. In addition, empagliflozin significantly increased the expression of Smad7, a known TGF-β1 and myocardial fibrosis inhibitor [288]. These benefits likely depend on a direct effect on human cardiac myofibroblasts, as an exposition of these cells to empagliflozin induce a more quiescent phenotype and a significantly attenuated cell-mediated extracellular matrix remodeling [292]. The regulation of Sestrin 2, a stress-inducible protein that regulates AMPK-mTOR and maintains redox homeostasis, is reported as a novel mechanism by which empagliflozin may improve obesity-related myocardial hypertrophy/fibrosis and cardiac dysfunction [293].

HFpEF, a frequent form of HF in diabetic patients, has been the subject of much research with SGLT2-Is. This condition is characterized by a special metabolic and pro-inflammatory status, accompanied by oxidative stress and disturbances of microvascular endothelial cells, whose normal activity greatly contributes to cardiomyocyte function and, when deranged, to HFpEF development [294]. In a co-culture of cardiac microvascular endothelial cells and cardiomyocytes, the stimulation of endothelial cells with TNF-α induced mitochondrial overproduction of ROS with decreased NO bioavailability and microvascular dysfunction with deterioration of cardiomyocyte systolic and diastolic function, all alterations restored by empagliflozin [295]. A hypophosphorylation of myofilament proteins may be the fault behind the increased LV stiffness, as observed in a study in myocardial tissue samples from an HFpEF dog model [296]. Empagliflozin was able to directly reduce the myofilament passive stiffness and improve diastolic function in murine and human HF myocardium by enhancing the phosphorylation level of titin and other myofilament regulatory proteins [297]. The involved mechanism has been identified in improving the NO-soluble guanylyl cyclase (sGC)-cGMP-PKG signaling observed in diabetic mice after 8 weeks of treatment with empagliflozin [298]. This result has been recently substantiated in more in-depth mechanistic research in vitro, showing that human and murine HFpEF myocardium treated with empagliflozin exhibited reduced markers of oxidative stress (H2O2, 3-nitrotyrosine, GSH, lipid peroxide) and inflammation (ICAM-1, VCAM-1, TNFα and IL-6). The consequent amelioration of NO levels, sGC activity, cGMP concentration, and PKG type Iα activity improved both endothelial vasorelaxation and phosphorylation of myofilament proteins with attenuated cardiomyocyte stiffness [299].

### 10.2. Evidence of Beneficial Anti-Inflammatory, Anti-Oxidant and Anti-Fibrotic Effects on Kidney

SGLT2-Is may halt the activation of inflammatory, oxidant, and profibrotic pathways involved in the pathogenesis of several functional and structural disorders of CKD [300].

A primary mechanism may rely on correcting enhanced glucose entry via SGLT2 transporter during hyperglycemia, as demonstrated in in vitro investigations in cultured human PTCs exposed to HG medium. The blockade of glucose reabsorption in PTCs by SGLT2-Is suppressed inflammation by inhibiting the NF-κB pathway and promoted autophagic flux via increased AMPK activity and mTOR inhibition [301,302]. In another study, HG and the associated increase of AGEs in PTCs suppressed the expression of the anti-fibrotic mediator RECK via induction of oxidative stress-dependent TRAF3IP2/NF-κB and p38 MAPK/miR-21 pathways, whereas treatment with empagliflozin ameliorated these alterations and inhibited the epithelial-to-mesenchymal transition and the tubule cell migration [303]. A study demonstrated that HG-treated PTCs presented an increased production of angiotensinogen in consequence of excess intracellular ROS, a peptide that may contribute to DKD development. This hyperproduction was limited by the blockade of SGLT2 [304].

In a model of T2DM mice, dapagliflozin attenuated the activation of NLPR3 inflammasome and the progression of DKD, as indicated by reduced renal expression of mRNA of collagens type 1 and type 3 [305]. Among the inflammatory molecules, IL-6 seems to be a key mediator for CKD development. The blockade of its receptor with Tocilizumab in T2DM mice blunted the activation of NLRP3 inflammasome and reduced both proteinuria and glomerular mesangial matrix accumulation [306].

Canagliflozin stimulated antioxidant/anti-inflammatory signaling pathways in rats treated with isoprenaline and prevented apoptosis of kidney cells by inhibiting Bax protein upregulation and caspase-3 activation. At the histological examination of kidney sections, attenuation in inflammatory cell infiltration, collagen deposition, and fibrosis were observed [307]. Other histopathological investigations showed beneficial changes by dapagliflozin in renal tissue from obese prediabetic rats, improving parameters related to inflammation, fibrosis, endoplasmic reticulum stress, and apoptosis [308]. In a recent study using a rat model of T2DM, dapagliflozin prevented the typical hystopathological lesions of DKD, i.e., glomerulomegaly, glomerular basement thickening, mesangial hypercellularity, and tubular damage [309]. The responsible mechanisms identified by immunohistochemical examination were a reduced expression in glomerular, mesangial, and tubular epithelial cells of α-smooth muscle actin, a hallmark of tubular epithelial-myofibroblast trans-differentiation and extracellular matrix deposition and glomerulosclerosis. The analysis of renal tissue homogenate showed an improvement of apoptotic markers (BCL-2 and BAX) and of TNF-α with a partial restoration of lowered renal expression of pentraxin 3 (PTX3), a factor that favorably attenuates the inflammatory activity of macrophages, promotes the M2 phenotype of these cells and downregulates NF-kB, IL-1β, TNF-α and monocyte chemoattractant protein 1 [310].

Consistent with what is described for the heart, activation of inflammation ultimately promotes tubulointerstitial fibrosis, a process blunted by SGLT2-Is. In T2DM and T2DM mouse models, dapagliflozin reduced mesangial expansion, macrophage infiltration, and interstitial fibrosis, through significant attenuation of STAT1 and TGFβ1 expression, both factors associated with tubulointerstitial fibrosis in DKD [119,311].

A transcriptomic analysis of plasma biomarkers from T2DM patients treated for 2 years with canagliflozin concerning those treated with glimepiride showed a decrease of genes encoding TNF receptor 1, IL-6, matrix metalloproteinase 7, and fibronectin 1, suggesting that SGLT2 inhibition may attenuate molecular processes typically related to DKD progression, such as inflammation, ECM turnover, and fibrosis [312]. At the same time, metabolomics analysis of kidney biopsy samples from patients with DKD treated with dapagliflozin demonstrated an upregulation of genes involved in the pathway of eNOS and endothelial function, in addition to molecular processes associated with energy metabolism and mitochondrial function [313].

## 11. Modulation of Mitochondrial Function and Autophagy by SGLT2-Is

### 11.1. Evidence in Heart

SGLT2-Is may multifariously modulate mitochondrial function [155].

As previously mentioned, these drugs increase the plasma levels of ketone bodies, which can enhance the oxidation efficiency of the mitochondrial coenzyme Q couple and the free energy of cytosolic ATP hydrolysis and redistribute sodium and calcium ions through binding with NHE and/or SMIT1 with a resulting amelioration of mitochondrial energy production [140,155].

Disorders of mitochondrial dynamics are associated with contraction dysfunction and damage of the myocardium until the appearance of lethal cardiac failure [314,315]. SGLT2-Is can control both mitochondrial fusion and fission. The first is a process that increases the energy production capacity by intensively fusing mitochondria under the strict control of mitofusion1 (Mfn1), mitofusion2 (Mfn2), and Opa1. Mitochondrial fission is the removal of damaged organelles by their division in smaller parts and subsequent mitophagy, a selective form of autophagy implicated in the maintenance of mitochondrial homeostasis and cell survival, under the regulation of Dynamin-related protein 1 (Drp1) and mitochondrial fission factor (Mff) [209,316]. Dapagliflozin suppressed prolonged ventricular repolarization in a rat model of metabolic syndrome through augmentation of mitochondrial energy production and normalization of Mfn1/Mfn2 ratio [317]. In infarcted diabetic rats, empagliflozin improved myocardial microvascular injury via an AMPK-dependent inhibition of cardiomyocyte mitochondrial fission and reduced the necrosis area by normalizing the size and number of mitochondria in non-necrotic tissue [318,319].

Large evidence supports the important role of autophagy, a lysosome-mediated degradative pathway, in maintaining cardiac homeostasis and adaptation to stress [320]. Autophagy-mediated clearance of damaged organelles reduces inflammasome activation and may in part explain the anti-inflammatory and anti-oxidant properties of SGLT2-Is [283,319]. Since treatment with SGLT2-Is mimics a state of nutrient deprivation, the activation of AMPK, the master regulator of cellular energy homeostasis, could be the mediating mechanism of autophagy and cardioprotection [321]. Accordingly, canagliflozin significantly inhibited the inflammatory response and the increased autophagy flow in immune cells by activating AMPK [322]. Similarly, empagliflozin enhanced autophagy at the cardiac level by activating AMPK in a model of early-stage diabetes [323]. Also, SGLT2 inhibition by empagliflozin in myocardial infarction mouse models with and without DM demonstrated a reduced infarct size and myocardial fibrosis mediated by inhibiting the autophagic flux in the cardiomyocytes [324]. In another study, empagliflozin protected the heart from doxorubicin toxicity through an integrated Beclin 1, TLR9, and SIRT3 network involving autophagy, oxidative stress, and mitochondria [325].

### 11.2. Evidence in Kidney

Altered mitochondrial dynamics plays an equally important role in the pathogenesis of DKD and other renal diseases, characterized by enhanced mitochondrial fragmentation and impaired autophagy of various cell types, particularly of those more vulnerable to mitochondrial dysfunction such as PTCs and podocytes [326]. In a rat model of mitochondrial dysfunction induced by a high-fat diet, ipragliflozin restored the renal tubular levels of Mfn2 and Opa1, controlling the mitochondrial fusion and those of the GTPases involved in mitochondrial biogenesis [327]. In human renal PTCs cultured under HG-conditions, empagliflozin reversed mitochondrial dynamics by improving the expression of proteins involved in mitochondrial fusion and fission and enhancing autophagy [328].

The marked impairment of capacity for autophagy in both podocytes and renal tubular cells in T2DM and other kidney diseases contributes to renal injury [329]. SGLT2-Is may correct this defect by inducing hypoxia-like transcriptional patterns that enhance SIRT1/HIF-2α signaling cells [330,331]. In obese diabetic mice, empagliflozin activated autophagy in podocytes, preventing their effacement, detachment, and death, thus reducing albuminuria [332].

## 12. Effects of SGLT2-Is on Erythropoietin and Erythropoiesis

All SGLT-2-Is have demonstrated a modest increase (2–4%) of hematocrit in their respective CVOTs, likely in dependence of hemoconcentration caused by enhanced diuresis. However, the different time course of change in urine volume and hematocrit supports other mechanisms, namely stimulation of erythropoiesis according to the concomitant increase in erythropoietin level and reticulocyte count during SGLT2-I treatment [333,334]. In a recent RCT, empagliflozin increased hemoglobin concentration and hematocrit after 3 months of treatment, which was most likely attributable to increased erythropoiesis rather than hemoconcentration, given the simultaneous increase in iron utilization [335]. Erythropoietin is produced by renal interstitial fibroblasts under the regulation of hypoxia-inducible factors [336]. The excessive glucose uptake via SGLT2 in diabetic patients overloads these cells and increases the mitochondrial oxygen consumption to meet the high demand to supply ATP for the Na+/K+ pump, resulting in renal cortex hypoxia [337]. Animal studies have shown that selective damage of PTCs induces trans-differentiation of erythropoietin-producing fibroblasts into myofibroblasts that actively synthesize fibrogenic molecules but have lost the ability to produce erythropoietin [338]. On this basis, the hypoxic microenvironment in the proximal tubules may explain why the serum erythropoietin is low in T2DM patients, even when kidney function is normal and further decreases as the glycosylated hemoglobin level increases [339].

By reducing glucose reabsorption, SGLT2-Is might reduce the metabolic stress of proximal tubules and improve tubulointerstitial hypoxia, thus inducing myofibroblasts to resume erythropoietin production. Ultimately, the increase of hematocrit during SGLT2-I therapy would indicate the recovery of tubulointerstitial function in the diabetic kidney [333].

According to another investigation, the increase of erythropoiesis by SGLT2 inhibition involves the suppression of hepcidin, a peptide that increases in pro-inflammatory conditions and inhibits erythropoiesis [340]. Other mechanisms operating in a state of SGLT2 inhibition might be the reduced afferent renal blood flow with decreased oxygen delivery, the increased but less efficient oxygen-consuming active sodium reabsorption at the distal tubule leading to the expression of hypoxia-inducible factors and the stimulation of erythropoietin by β-HB [341]. On the other hand, among the pathways whereby SGLT2-Is exert their nephroprotective effects in diabetic patients, the improved tissue oxygen tension within the renal cortex has been taken into account, which is not congruent with enhanced production of erythropoietin. In a recent in vivo study in diabetic rats treated with dapagliflozin, a significant reduction in microvascular kidney oxygen tension was demonstrated in the deeper cortex and outer medulla, right where SGLT1 are primarily located, but not in the superficial cortex that includes few if any S3 segments. Consistent with this observation, hypoxia-responsive erythropoietin mRNA levels were elevated, and reticulocyte counts increased [342]. These findings likely reflect the interplay of the TGF-induced afferent arteriolar constriction, decreased sodium reabsorption in the early proximal tubule, and increased sodium reabsorption at the S3 segment PTCs.

The enhanced erythropoiesis by SGLT2-Is is observed in diabetic patients with both normal or reduced kidney function [343]. Among patients with CKD, SGLT2 inhibition was associated with a reduced risk of anemia-associated outcomes, including the need for erythropoiesis-stimulating agents [344].

The elevation of erythropoietin may be among the mechanisms mediating the nephroprotection by SGLT2-Is, as supported by observations in animal models of DKD [345]. Similarly, the increased red cell mass may contribute to improved myocardial tissue oxygen supply and reduced LV mass in diabetic patients with coronary artery disease [341,346]. In post-hoc mediation analyses of data from the EMPAREG OUTCOME trial, it was estimated that the change of hematocrit and hemoglobin respectively mediated 51% and 54% of the risk reduction of CV death that mainly depends on the reduction of HF events [347].

## 13. Concluding Remarks and Future Perspectives

In the past few years, evidence from large RCTs revealed the unexpected but powerful and indisputable cardiorenal protective properties of SGLT2-Is, well beyond their initial therapeutic target of lowering glucose, that occur early after the start of treatment mainly due to its non-glycemic effects and is therefore efficacious even in patients without diabetes. Based on these benefits, for the first time in the history of diabetology, the guidelines of diabetes have undergone an epochal change for which treatment adjustments are not dictated primarily by HbA1c value, but rather by the coexistence of or risk for kidney or cardiovascular disease. And again, for the first time, medicaments intended for the treatment of diabetes were included in the therapeutic equipment of heart failure. These features rendered these drugs unique in the current therapeutic landscape of diabetes and HF.

In recent years, we have witnessed a huge effort of scientific research attempting to identify the mechanisms underlying the sparkling results of clinical trials, and the field remains in active ferment. Certain aspects have been clearly defined, such as the nephroprotection relying mainly on the target action of SGLT2 inhibition that generates reduced glucose reabsorption and modulation of afferent arteriole tone, interfering with the major pathophysiological culprits of proteinuric CKD progression, i.e., glomerular hypertension and hyperfiltration. Instead, it is amazing how inhibitors of SGLT2 can exert strong protection of the heart, of even greater magnitude than on the kidney and comparable to that of existing anti-heart failure drugs, without SGLT2 being expressed in cardiomyocytes. The modulation of sodium homeostasis and some other effects appear to result from actions carried out directly at the myocardial level. Otherwise, the literature has been enriched with an expanding plethora of pathophysiological theories that prioritize metabolic, diuretic, hemodynamic, or other changes and involve multi-factorial, complementary, and intertwined pathways. However, since it is unlikely that a class of drugs interacts with a myriad of specific targets, it is more reasonable to speculate that SGLT2 inhibitors influence, if not just one, only some upstream targets in the pathophysiology of heart failure. Thus, the mechanistic details of the SGLT-2 inhibitor action need further investigation.

Based on the impressive benefits on cardiorenal protection that emerged from trials, SGLT2-Is deserve to be specifically explored as potential therapeutic means against the so-called “cardiorenal syndrome”, a term coined about twenty years ago to define the strong interaction between HF and renal disorders, as already known for two centuries. Likely, the medications may counteract the development of this syndrome through the correction of strong risk factors such as diabetes and hypertension, and some well-established pathophysiological mechanisms such as oxidative stress and inflammation.

Many other uncertainties need to be removed, as an example to disclose if the effects are class-driven or molecule-specific and if other pathologies can benefit from therapy, such as type 1 diabetes mellitus, nonalcoholic steatohepatitis, or obesity, as well as non-diabetic chronic kidney diseases. Crucially, it is unknown whether the benefit extends over a long period or involves protection against atherosclerotic cardiovascular disease and if the therapy can not only attenuate but even prevent cardiac and renal damage. This data will help identify those patients who can benefit most from these drugs, and hopefully extend their use to as many people as possible. In this regard, clinicians must be aware that SGLT2-Is may be used, paying great attention to patients with moderate CKD, and that adverse events, such as UTIs, occur more frequently in elderly patients. Likewise, it is crucial that in ketosis-prone conditions, including alcoholism, ketogenic diet, and prolonged fasting states for any reason, gliflozins are administered under strict monitoring and after previous patients training.

## Figures and Tables

**Table 1:** Main clinical trials evaluating cardiovascular outcomes among patients treated with SGLT2-Is.

Drug	Trial (Ref.)	Patients	Follow-Up (Median)	Outcomes	Hazard Ratio (95% CI)
**Empagliflozin**	**EMPAREG OUTCOME study [47]**	**7020 T2DM patients at high risk for CV events and an eGFR ≥ 30 mL/min/1.73 m^2^**	**3.1 years** (2.6 years of treatment)	**composite of death from CV causes, nonfatal myocardial infarction, or nonfatal stroke**	**0.86** (0.74–0.99)
		**empagliflozin 10 mg vs.** **empagliflozin 25 mg vs.** **matching placebo**		**death from cardiovascular causes** **nonfatal myocardial infarction** **nonfatal stroke**	**0.62** (0.49–0.77) 0.87 (0.70–1.09) 1.24 (0.92–1.56)
				**hospitalization for heart failure**	**0.65** (0.50–0.85)
				**death from any cause**	**0.68** (0.57–0.82)
	**EMPEROR-Reduced [101]**	**3730 diabetic or not diabetic patients with class II, III, or IV HF and EF ≤ 40%**	**16 months**	**cardiovascular death or hospitalization for worsening heart failure**	**0.75** (0.65–0.86)
		**empagliflozin 10 mg vs. placebo** **(in addition to recommended therapy)**		**hospitalization for heart failure**	**0.70** (0.58–0.85)
	**EMPEROR-Preserved [114]**		**26.2 months**	**cardiovascular death or hospitalization for worsening heart failure**	**0.79** (0.69–0.90)
		**5988 diabetic or not diabetic patients with class II-IV HF and EF > 40%**		**hospitalization for heart failure**	**0.73** (0.61–0.88)
		**empagliflozin 10 mg vs. placebo** **(in addition to usual therapy)**			
	**EMPERIAL [104]**		**12 weeks**	**change in 6-minute walk test distance**	*ns*
		**patients with HFrE (EF ≤ 40%, *n* = 312) or with HFpEF (EF > 40%, *n* = 315)**		**KCCQ-TSS ** (Kansas City Cardiomyopathy Questionnaire Total Symptom Score)	*ns*
		**empagliflozin 10 mg vs. placebo**		**CHQ-SAS** (Chronic Heart Failure Questionnaire Self-Administered Standardized format) dyspnoea score	*ns*
**Canagliflozin**	**CANVAS study [46]**	**10,142 participants with T2DM and high CV risk** **100 mg (with an optional increase to 300 mg) vs. placebo**	**188.2 weeks**	**composite of death from CV causes, nonfatal myocardial infarction, or nonfatal stroke**	**0.86** (0.75–0.97)
**Dapagliflozin**	**DECLARE-TIMI 58 [48]**	**17,160 T2DM patients at high risk for CV events (only 7% of patients had an eGFR < 60 mL/min/1.73 m^2^)**	**4.2 years**	**composite of CV death, myocardial infarction, or ischemic stroke**	0.93 (0.84–1.03)
		**dapagliflozin 10 mg vs. placebo**		**CV death or hospitalization for HF**	**0.83** (0.73–0.95)
				**hospitalization for HF**	**0.73** (0.61–0.88)
	**DAPA-HF Trial [100]**	**4304 diabetic (68%) or not diabetic patients with class II-IV HF**	**18.2 months**	**worsening HF (hospitalization or urgent visit resulting in IV therapy for HF) or CV death**	**0.74** (0.65–0.85)
		**dapagliflozin 10 mg vs. placebo**		**first worsening HF event**	**0.70** (0.59–0.83)
		**(in addition to recommended therapy)**		**CV death**	**0.83** (0.71–0.97)
	**DEFINE HF [103]**	**263 diabetic or not diabetic patient with class II-III HF**	**12 weeks**	**mean NT-proBNP**	***ns***
		**dapagliflozin 10 mg vs. placebo**		**% of patients with ameliorated functional status**	**1.8** (1.03–3.06)
**Sotagliflozin**	**SCORED [93]**	**10,584 T2DM patients with CKD and CV risk** **sotagliflozin 200–400 mg vs. placebo**	**16 months**	**composite of CV death, hospitalization fo HF, and urgent visit for HF**	**0.74** (0.63–0.88)
	**SOLOIST-WHF [102]**	**1222 T2DM patients recently hospitalized for worsening HF** **sotagliflozin 200–400 mg vs. placebo**	**9 months**	**CV deaths and hospitalization or urgent visits for HF**	**0.67** (0.52–0.85)
				**CV death**	0.84 (0.58–1.22)
				**death from any cause**	0.82 (0.59–1.14)
**Ertugliflozin**	**VERTIS CV study [92]**	**8246 T2DM patients with established CV disease and an eGFR ≥ 30 mL/min/ 1.73 m^2^**	**3 years**	**composite of CV death, myocardial infarction, or ischemic stroke**	**0.97** (0.85–1.11)
		**ertugliflozin 5 or 15 mg vs. placebo**		**death from CV causes or hospitalization for HF**	**0.88** (0.75–1.03)
				**death from CV causes **	**0.92** (0.77–1.11)

**Table 2:** Main clinical trials evaluating renal outcomes among patients treated with SGLT2-Is.

Drug	Trial (Ref.)	Patients	Follow-Up (Median)	Outcomes	Hazard Ratio (95% CI)
**Empagliflozin**	**EMPAREG OUTCOME study [47]**	**7020 T2DM patients with high risk for CV events and eGFR ≥ 30 mL/min/1.73 m^2^**	**3.1 years** (2.6 years of treatment)	**incident or worsening nephropathy**	**0.61** (0.53–0.70)
				**progression to macroalbuminuria**	**0.65** (0.54–0.72)
		**empagliflozin 10 mg vs.**		**doubling of the serum creatinine level**	**0.56** (0.39–0.79)
		**empagliflozin 25 mg vs.** **placebo**		**initiation of renal-replacement therapy**	**0.45** (0.21–0.97)
				**post hoc composite of doubling of serum creatinine, renal replacement therapy, or death for renal causes**	**0.54** (0.40–0.75)
				**incident albuminuria**	**0.95** (0.87–1.04)
**Canagliflozin**	**CANVAS–R study [46]**	**5812 T2DM patients with high risk for CV events and eGFR > 30 mL/min/1.73 m^2^**	**126.1 weeks**	**lower progression of albuminuria**	**0.73** (0.67–0.79)
		**canagliflozin 100 or 300 mg vs. placebo**		**composite of 40% reduction in eGFR, renal replacement therapy, or death from renal causes**	**0.60** (0.47–0.77)
	**CREDENCE study [105]**	**4401 T2DM patients with albuminuric CKD (eGFR of 30 to < 90 mL/min/1.73 m^2^)**	**2.62 years**	**composite of ESRD** (dialysis, transplantation, or sustained eGFR < 15 mL/min/1.73 m^2^), **doubling of serum creatinine, or death from renal or CV causes**	**0.70** (0.59–0.82)
		**canagliflozin 100 mg vs. placebo**		**composite of ESRD, a doubling of the creatinine level, or death from renal causes**	**0.66** (0.53–0.81)
				**composite of cardiovascular death, myocardial infarction, or stroke**	**0.80** (0.67–0.95)
				**hospitalization for heart failure**	**0.61** (0.47–0.80)
**Dapagliflozin**	**DECLARE-TIMI 58 [48]**	**7160 T2DM patients at high risk for CV events (only 7% with eGFR < 60 mL/min/1.73 m^2^)** **dapagliflozin 10 mg vs. placebo**	**4.2 years**	**composite of ≥40% reduction in eGFR, new ESRD, or death from renal or CV causes**	**0.76** (0.67–0.87)
	**DAPA-CKD study [106]**	**4304 diabetic (68%) or not diabetic patients suffering from CKD** (UACR 200–5000 mg/g and eGFR 25–75 mL/min/1.73 m^2^)	**2.4 years**	**composite of** **≥50% sustained decline in eGFR or ESRD or CV or renal death**	**0.56** (0.45–0.69)
		**dapagliflozin 10 mg vs. placebo**		**composite of CV death and hospitalization for heart failure**	**0.71** (0.55–0.92)
	**DELIGHT study [107]**	**461 T2DM patients with albuminuria** (UACR 30–3500 mg/g) **and eGFR of 25–75 mL/min/1.73 m^2^, treated with ACE-Is or ARBs**	**24 weeks**	**variation of albumin-to-creatinine ratio**	−21.0% for dapagliflozin (*p* = 0.011)
		**dapagliflozin 10 mg vs.** **dapagliflozin 10 mg–saxagliptin 2.5 mg vs.** **placebo**			−38.0% for dapagliflozin + saxagliptin (<0.0001)
	**DERIVE study [108]**	**321 T2DM patients with CKD in stage 3A (eGFR of 45–59 mL/min/1.73 m^2^)** **dapagliflozin 10 mg vs. placebo**	**24 weeks**	**change from baseline in urine eGFR**	decrease at week 4 with a trend to recovery at weeks 12 and 24 eGFR similar to placebo after a 3 week period without treatment
	**DIAMOND study [109]**	**53 non-diabetic patients with CKD** (24-h urinary protein excretion > 500 mg and ≤3500 mg, eGFR ≥ 25 mL/min/1.73 m^2^) **on stable RAS blockade** **27 received dapagliflozin 10 mg then placebo** **26 received placebo then dapagliflozin 10 mg**	**cross-over trial** (6 weeks for each treatment and washout period)	**mean proteinuria** **measured GFR**	**no significant change from baseline** **change with dapagliflozin at week 6 by −6.6 mL/min/1.73 m^2^** **(−9.0 to −4.2; *p* < 0.0001)** (fully reversible within 6 weeks after dapagliflozin discontinuation)
**Ertugliflozin**	**VERTIS CV study [92]**	**8246 T2DM patients with established CV disease and eGFR ≥ 30 mL/min/1.73 m^2^** **ertugliflozin 5 or 15 mg vs. placebo**	**3 years**	**composite of death from renal causes, renal replacement therapy, or doubling of serum creatinine **	**0.81** (0.63–1.04)

## Abbreviations

AGE products: advanced glycation end products; AMPK: AMP-activated protein kinase; BCAAs: branched chain amino acids; BP: blood pressure; BW: body weight; CaMKII: Ca^2+^/Calmodulin-dependent kinase II; CKD: chronic kidney disease; CV: cardiovascular; CVD: cardiovascular disease; CVOTs: cardiovascular outcome trials; DKA: diabetic ketoacidosis; DKD: diabetic kidney disease; eGFR: estimated glomerular filtration rate; FA: fatty acid; GFR: glomerular filtration rate; GLP-1: glucagon-like peptide 1; HFpHF: HF with preserved ejection fraction; HFrEF: HF with reduced ejection fraction; β-HB: β-hydroxybutyrate; HIF-1α: hypoxia-inducible factor 1α; HG: high glucose; HNF-1α: hepatocyte nuclear factor-1α; I_NaL_: late sodium current; LV: left ventricle; MACEs: major adverse cardiovascular events; MAPK: mitogen-activated protein kinases; NCX: sarcolemmal Na^+^/Ca^2+^ exchanger; NCLX: mitochondrial Na^+^/Ca^2+^ exchanger; NF-kB: nuclear factor κB; NHE1: sodium-hydrogen exchanger 1; NHE3: sodium-hydrogen exchanger 3; NLRP3: nucleotide-binding domain-like receptor protein 3; NOS: NO synthase; NOX: NADPH oxidases; Nrf2: nuclear factor erythroid 2; PKG: protein kinase G; P/O: ATP production/O_2_ consumption; PTCs: proximal tubule cells; PKG: protein kinase G; RCT: randomized controlled trial; RAAS: renin-angiotensin-aldosterone system; RAGE: receptor for AGE; ROS: reactive oxygen species; SCOT: succinyl-CoA:3-ketoacid CoA transferase; sGC: soluble guanylyl cyclase; SGLT2: sodium-glucose linked transporter 2; SGLT2-Is: SGLT2 inhibitors; SMIT1: sodium–myoinositol cotransporter-1; sGC: soluble guanylyl cyclase; T1DM: type 1 diabetes mellitus; T2DM: type 2 diabetes mellitus; TGF: tubuloglomerular feedback; TGFβ1: transforming growth factor β1; UTIs: urinary tract infectious.

## References

[1] Salvatore T., Carbonara O., Cozzolino D., Torella R., Nasti R., Lascar N., Sasso F.C. (2011). Kidney in diabetes: From organ damage target to therapeutic target. Curr. Drug Metab..

[2] DeFronzo R.A., Hompesch M., Kasichayanula S., Liu X., Hong Y., Pfister M., Morrow L.A., Leslie B.R., Boulton D.W., Ching A. (2013). Characterization of renal glucose reabsorption in response to dapagliflozin in healthy subjects and subjects with type 2 diabetes. Diabetes Care.

[3] Merovci A., Mari A., Solis-Herrera C., Xiong J., Daniele G., Chavez-Velazquez A., Tripathy D., Urban McCarthy S., Abdul-Ghani M., DeFronzo R.A. (2015). Dapagliflozin lowers plasma glucose concentration and improves β-cell function. J. Clin. Endocrinol. Metab..

[4] De Konink L. (1836). Observations sur les proprietes febrifuges de la phloridzine. Bull. Soc. Med. Gand.

[5] Chasis H., Jolliffe N., Smith H.W. (1933). The action of phlorizin on the excretion of glucose, xylose, sucrose, creatinine and urea by man. J. Clin. Investig..

[6] Galiero R., Pafundi P.C., Nevola R., Rinaldi L., Acierno C., Caturano A., Salvatore T., Adinolfi L.E., Costagliola C., Sasso F.C. (2020). The Importance of Telemedicine during COVID-19 Pan-demic: A Focus on Diabetic Retinopathy. J. Diabetes Res..

[7] Lombardi R., Airaghi L., Targher G., Serviddio G., Maffi G., Mantovani A., Maffeis C., Colecchia A., Villani R., Rinaldi L. (2020). Liver fibrosis by FibroScan® independently of established cardiovascular risk parameters associates with macrovascular and microvascular complications in patients with type 2 diabetes. Liver Int..

[8] Marfella R., Sasso F.C., Cacciapuoti F., Portoghese M., Rizzo M.R., Siniscalchi M., Carbonara O., Ferraraccio F., Torella M., Petrella A. (2012). Tight glycemic control may increase regenerative potential of myocardium during acute infarction. J. Clin. Endocrinol. Metab..

[9] Sasso F.C., Rinaldi L., Lascar N., Marrone A., Pafundi P.C., Adinolfi L.E., Marfella R. (2018). Role of Tight Glycemic Control during Acute Coronary Syndrome on CV Outcome in Type 2 Diabetes. J. Diabetes Res..

[10] Caturano A., Galiero R., Pafundi P.C., Cesaro A., Vetrano E., Palmiero G., Rinaldi L., Salvatore T., Marfella R., Sardu C. (2021). Does a strict glycemic control during acute coronary syndrome play a cardioprotective effect? Pathophysiology and clinical evidence. Diabetes Res. Clin. Pract..

[11] Kang A., Jardine M.J. (2021). SGLT2 inhibitors may offer benefit beyond diabetes. Nat. Rev. Nephrol..

[12] Fathi A., Vickneson K., Singh J.S. (2021). SGLT2-inhibitors; more than just glycosuria and diuresis. Heart Fail Rev..

[13] Thomas M.C., Cherney D.Z.I. (2018). The actions of SGLT2 inhibitors on metabolism, renal function and blood pressure. Diabetologia.

[14] Vallon V., Thomson S.C. (2017). Targeting renal glucose reabsorption to treat hyperglycaemia: The pleiotropic effects of SGLT2 inhibition. Diabetologia.

[15] Merovci A., Solis-Herrera C., Daniele G., Eldor R., Fiorentino T.V., Tripathy D., Xiong J., Perez Z., Norton L., Abdul-Ghani M.A. (2014). Dapagliflozin improves muscle insulin sensitivity but enhances endogenous glucose production. J. Clin. Investig..

[16] Hu S., Lin C., Cai X., Zhu X., Lv F., Nie L., Ji L. (2022). The Urinary Glucose Excretion by Sodium-Glucose Cotransporter 2 Inhibitor in Patients With Different Levels of Renal Function: A Systematic Review and Meta-Analysis. Front. Endocrinol..

[17] van Bommel E.J., Muskiet M.H., Tonneijck L., Kramer M.H., Nieuwdorp M., van Raalte D.H. (2017). SGLT2 Inhibition in the Diabetic Kidney-From Mechanisms to Clinical Outcome. Clin. J. Am. Soc. Nephrol..

[18] Ferrannini E., Muscelli E., Frascerra S., Baldi S., Mari A., Heise T., Broedl U.C., Woerle H.J. (2014). Metabolic response to sodium-glucose cotransporter 2 inhibition in type 2 diabetic patients. J. Clin. Investig..

[19] Foster D.W. (2012). Malonyl-CoA: The regulator of fatty acid synthesis and oxidation. J. Clin. Investig..

[20] Zambrowicz B., Freiman J., Brown P.M., Frazier K.S., Turnage A., Bronner J., Ruff D., Shadoan M., Banks P., Mseeh F. (2012). LX4211, a dual SGLT1/SGLT2 inhibitor, improved glycemic control in patients with type 2 diabetes in a randomized, placebo-controlled trial. Clin. Pharmacol. Ther..

[21] Cherukuri L., Smith M.S., Tayek J.A. (2018). The durability of oral diabetic medications: Time to A1c baseline and a review of common oral medications used by the primary care provider. Endocrinol. Diabetes Metab. J..

[22] Vaduganathan M., Inzucchi S.E., Sattar N., Fitchett D.H., Ofstad A.P., Brueckmann M., George J.T., Verma S., Mattheus M., Wanner C. (2021). Effects of empagliflozin on insulin initiation or intensification in patients with type 2 diabetes and cardiovascular disease: Findings from the EMPA-REG OUTCOME trial. Diabetes Obes. Metab..

[23] Yang Y., Chen S., Pan H., Zou Y., Wang B., Wang G., Zhu H. (2017). Safety and efficiency of SGLT2 inhibitor combining with insulin in subjects with diabetes: Systematic review and meta-analysis of randomized controlled trials. Medicine.

[24] Mirabelli M., Chiefari E., Caroleo P., Vero R., Brunetti F.S., Corigliano D.M., Arcidiacono B., Foti D.P., Puccio L., Brunetti A. (2019). Long-Term Effectiveness and Safety of SGLT-2 Inhibitors in an Italian Cohort of Patients with Type 2 Diabetes Mellitus. J. Diabetes Res..

[25] Bailey C.J., Del Prato S., Wei C., Reyner D., Saraiva G. (2019). Durability of glycaemic control with dapagliflozin, an SGLT2 inhibitor, compared with saxagliptin, a DPP4 inhibitor, in patients with inadequately controlled type 2 diabetes. Diabetes Obes. Metab..

[26] Wysham C.H., Lefebvre P., Pilon D., Lafeuille M.H., Emond B., Kamstra R., Pfeifer M., Duh M.S., Ingham M. (2019). An investigation into the durability of glycemic control in patients with type II diabetes initiated on canagliflozin or sitagliptin: A real-world analysis of electronic medical records. J. Diabetes Complicat..

[27] Dimitrakoudis D., Vranic M., Klip A. (1992). Effects of hyperglycemia on glucose transporters of the muscle: Use of the renal glucose reabsorption inhibitor phlorizin to control glycemia. J. Am. Soc. Nephrol..

[28] Jurczak M.J., Lee H.-Y., Birkenfeld A.L., Jornayvaz F.R., Frederick D.W., Pongratz R.L., Zhao X., Moeckel G.W., Samuel V.T., Whaley J. (2011). SGLT2 deletion improves glucose homeostasis and preserves pancreatic beta-cell function. Diabetes.

[29] Obata A., Kubota N., Kubota T., Iwamoto M., Sato H., Sakurai Y., Takamoto I., Katsuyama H., Suzuki Y., Fukazawa M. (2016). Tofogliflozin Improves Insulin Resistance in Skeletal Muscle and Accelerates Lipolysis in Adipose Tissue in Male Mice. Endocrinology.

[30] Merovci A., Abdul-Ghani M., Mari A., Solis-Herrera C., Xiong J., Daniele G., Tripathy D., DeFronzo R.A. (2016). Effect of Dapagliflozin With and Without Acipimox on Insulin Sensitivity and Insulin Secretion in T2DM Males. J. Clin. Endocrinol. Metab..

[31] Li D., Zou H., Yin P., Li W., He J., Wang S., Huang L., Shao S., Chen Y., Yang Y. (2021). Durability of glycaemic control in type 2 diabetes: A systematic review and meta-analysis for its association with body weight changes. Diabetes Obes. Metab..

[32] Meier J.J., Nauck M.A. (2005). Glucagon-like peptide 1(GLP-1) in biology and pathology. Diabetes Metab. Res. Rev..

[33] Frías J.P., Guja C., Hardy E., Ahmed A., Dong F., Öhman P., Jabbour S.A. (2016). Exenatide once weekly plus dapagliflozin once daily versus exenatide or dapagliflozin alone in patients with type 2 diabetes inadequately controlled with metformin monotherapy (DURATION-8): A 28 week, multicentre, double-blind, phase 3, randomised controlled trial. Lancet Diabetes Endocrinol..

[34] Patoulias D., Stavropoulos K., Imprialos K., Katsimardou A., Kalogirou M.S., Koutsampasopoulos K., Zografou I., Papadopoulos C., Karagiannis A., Doumas M. (2019). Glycemic efficacy and safety of glucagon-like peptide-1 receptor agonist on top of sodium-glucose co-transporter-2 inhibitor treatment compared to sodium-glucose co-transporter-2 inhibitor alone: A systematic review and meta-analysis of randomized controlled trials. Diabetes Res. Clin. Pract..

[35] Li D., Shi W., Wang T., Tang H. (2018). SGLT2 inhibitor plus DPP-4 inhibitor as combination therapy for type 2 diabetes: A systematic review and meta-analysis. Diabetes Obes. Metab..

[36] Min S.H., Yoon J.H., Hahn S., Cho Y.M. (2017). Comparison between SGLT2 inhibitors and DPP4 inhibitors added to insulin therapy in type 2 diabetes: A systematic review with indirect comparison meta-analysis. Diabetes Metab. Res. Rev..

[37] Yang Y., Zhao C., Ye Y., Yu M., Qu X. (2020). Prospect of Sodium-Glucose Co-transporter 2 Inhibitors Combined With Insulin for the Treatment of Type 2 Diabetes. Front. Endocrinol..

[38] Lu J., Tang L., Meng H., Zhao J., Liang Y. (2019). Effects of sodium-glucose cotransporter (SGLT) inhibitors in addition to insulin therapy on glucose control and safety outcomes in adults with type 1 diabetes: A meta-analysis of randomized controlled trials. Diabetes. Metab. Res. Rev..

[39] Forst T., Heise T., Plum-Morschel L. (2016). Pharmacological Intervention in Type 2 Diabetes Mellitus—A Pathophysiologically Reasoned Approach?. Curr. Diabetes Rev..

[40] Liao H.W., Wu Y.L., Sue Y.M., Lee M., Ovbiagele B. (2018). Sodium-glucose cotransporter 2 inhibitor plus pioglitazone vs. pioglitazone alone in patients with diabetes mellitus: A systematic review and meta-analysis of randomized controlled trials. Endocrinol. Diabetes Metab..

[41] Pelletier R., Ng K., Alkabbani W., Labib Y., Mourad N., Gamble J.M. (2021). Adverse events associated with sodium glucose co-transporter 2 inhibitors: An overview of quantitative systematic reviews. Ther. Adv. Drug Saf..

[42] Goldenberg R.M., Berard L.D., Cheng A.Y., Gilbert J.D., Verma S., Woo V.C., Yale J.-F. (2016). SGLT2 Inhibitor-associated Diabetic Ketoacidosis: Clinical Review and Recommendations for Prevention and Diagnosis. Clin. Ther..

[43] Bonner C., Kerr-Conte J.J., Gmyr V.V., Queniat G.G., Moerman E.E., Thévenet J.J., Beaucamps C.C., Delalleau N.N., Popescu I.I., Malaisse W.J. (2015). Inhibition of the glucose transporter SGLT2 with dapagliflozin in pancreatic alpha cells triggers glucagon secretion. Nat. Med..

[44] Garofalo C., Borrelli S., Liberti M.E., Andreucci M., Conte G., Minutolo R. (2019). SGLT2 Inhibitors: Nephroprotective Efficacy and Side Effects. Medicina.

[45] Kamei J., Yamamoto S. (2021). Complicated urinary tract infections with diabetes mellitus. J. Infect. Chemother..

[46] Neal B., Perkovic V., Mahaffey K.W., de Zeeuw D., Fulcher G., Erondu N., Shaw W., Law G., Desai M., Matthews D.R. (2017). Canagliflozin and Cardiovascular and Renal Events in Type 2 Diabetes. N. Engl. J. Med..

[47] Zinman B., Wanner C., Lachin J.M., Fitchett D., Bluhmki E., Hantel S., Mattheus M., Devins T., Johansen O.E., Woerle H.J. (2015). Empagliflozin, Cardiovascular Outcomes, and Mortality in Type 2 Diabetes. N. Engl. J. Med..

[48] Wiviott S.D., Raz I., Bonaca M.P., Mosenzon O., Kato E.T., Cahn A., Silverman M.G., Zelniker T.A., Kuder J.F., Murphy S.A. (2019). Dapagliflozin and Cardiovascular Outcomes in Type 2 Diabetes. N. Engl. J. Med..

[49] Chang H.Y., Singh S., Mansour O., Baksh S., Alexander G.C. (2018). Association Between Sodium-Glucose Cotransporter 2 Inhibitors and Lower Extremity Amputation Among Patients With Type 2 Diabetes. JAMA Intern. Med..

[50] Ueda P., Svanström H., Melbye M., Eliasson B., Svensson A.-M., Franzén S., Gudbjörnsdottir S., Hveem K., Jonasson C., Pasternak B. (2018). Sodium glucose cotransporter 2 inhibitors and risk of serious adverse events: Nationwide register based cohort study. BMJ.

[51] Gerich J.E. (2010). Role of the kidney in normal glucose homeostasis and in the hyperglycaemia of diabetes mellitus: Therapeutic implications. Diabet Med..

[52] Tahrani A.A., Barnett A.H., Bailey C.J. (2013). SGLT inhibitors in management of diabetes. Lancet Diabetes Endocrinol..

[53] DeFronzo R.A., Norton L., Abdul-Ghani M. (2017). Renal, metabolic and cardiovascular considerations of SGLT2 inhibition. Nat. Rev. Nephrol..

[54] Scheepers A., Joost H.G., Schürmann A. (2004). The glucose transporter families SGLT and GLUT: Molecular basis of normal and aberrant function. JPEN J. Parenter Enter. Nutr..

[55] Poulsen S.B., Fenton R.A., Rieg T. (2015). Sodium-glucose cotransport. Curr. Opin. Nephrol. Hypertens..

[56] Hediger M.A., Kanai Y., You G., Nussberger S. (1995). Mammalian ion-coupled solute transporters. J. Physiol..

[57] Wright E.M., Loo D.D., Hirayama B.A. (2011). Biology of human sodium glucose transporters. Physiol. Rev..

[58] Coady M.J., El Tarazi A., Santer R., Bissonnette P., Sasseville L.J., Calado J., Lussier Y., Dumayne C., Bichet D.G., Lapointe J.Y. (2017). MAP17 Is a Necessary Activator of Renal Na^+^/Glucose Cotransporter SGLT2. J. Am. Soc. Nephrol..

[59] DeFronzo R.A., Reeves W.B., Awad A.S. (2021). Pathophysiology of diabetic kidney disease: Impact of SGLT2 inhibitors. Nat. Rev. Nephrol..

[60] Thomson S.C., Vallon V. (2019). Renal Effects of Sodium-Glucose Co-Transporter Inhibitors. Am. J. Cardiol..

[61] Wilcox C.S. (2020). Antihypertensive and Renal Mechanisms of SGLT2 (Sodium-Glucose Linked Transporter 2) Inhibitors. Hypertension.

[62] Inoue B.H., dos Santos L., Pessoa T.D., Antonio E.L., Pacheco B.P., Savignano F.A., Carraro-Lacroix L.R., Tucci P.J., Malnic G., Girardi A.C. (2012). Increased NHE3 abundance and transport activity in renal proximal tubule of rats with heart failure. Am. J. Physiol. Integr. Comp. Physiol..

[63] Onishi A., Fu Y., Darshi M., Crespo-Masip M., Huang W., Song P., Patel R., Kim Y.C., Nespoux J., Freeman B. (2019). Effect of renal tubule-specific knockdown of the Na^+^/H^+^ exchanger NHE3 in Akita diabetic mice. Am. J. Physiol. Physiol..

[64] Pessoa T.D., Campos L.C., Carraro-Lacroix L., Girardi A.C., Malnic G. (2014). Functional role of glucose metabolism, osmotic stress, and sodium-glucose cotransporter isoform-mediated transport on Na^+^/H^+^ exchanger isoform 3 activity in the renal proximal tubule. J. Am. Soc. Nephrol..

[65] Wright E.M., Hirayama B.A., Loo D.F. (2007). Active sugar transport in health and disease. J. Intern. Med..

[66] Lee Y.J., Lee Y.J., Han H.J. (2007). Regulatory mechanisms of Na(+)/glucose cotransporters in renal proximal tubule cells. Kidney Int..

[67] Ghezzi C., Loo D.D.F., Wright E.M. (2018). Physiology of renal glucose handling via SGLT1, SGLT2 and GLUT2. Diabetologia.

[68] Jurczak M.J., Saini S., Ioja S., Costa D.K., Udeh N., Zhao X., Whaley J.M., Kibbey R.G. (2018). SGLT2 knockout prevents hyperglycemia and is associated with reduced pancreatic β-cell death in genetically obese mice. Islets.

[69] Powell D.R., Dacosta C.M., Gay J., Ding Z.-M., Smith M., Greer J., Doree D., Jeter-Jones S., Mseeh F., Rodriguez L.A. (2013). Improved glycemic control in mice lacking Sglt1 and Sglt2. Am. J. Physiol. Metab..

[70] Song P., Huang W., Onishi A., Patel R., Kim Y.C., Van Ginkel C., Fu Y., Freeman B., Koepsell H., Thomson S.C. (2019). Knockout of Na^+^-glucose cotransporter SGLT1 mitigates diabetes-induced upregulation of nitric oxide synthase NOS1 in the macula densa and glomerular hyperfiltration. Am. J. Physiol. Physiol..

[71] Vallon V. (2011). The proximal tubule in the pathophysiology of the diabetic kidney. Am. J. Physiol. Integr. Comp. Physiol..

[72] Freitas H.S., Anhê G.F., Melo K.F.S., Okamoto M.M., Oliveira-Souza M., Bordin S., Machado U.F. (2008). Na^+^-Glucose Transporter-2 Messenger Ribonucleic Acid Expression in Kidney of Diabetic Rats Correlates with Glycemic Levels: Involvement of Hepatocyte Nuclear Factor-1α Expression and Activity. Endocrinology.

[73] Vallon V., Gerasimova M., Rose M.A., Masuda T., Satriano J., Mayoux E., Koepsell H., Thomson S.C., Rieg T. (2014). SGLT2 inhibitor empagliflozin reduces renal growth and albuminuria in proportion to hyperglycemia and prevents glomerular hyperfiltration in diabetic Akita mice. Am. J. Physiol. Physiol..

[74] Kaur P., Behera B.S., Singh S., Munshi A. (2021). The pharmacological profile of SGLT2 inhibitors: Focus on mechanistic aspects and pharmacogenomics. Eur. J. Pharmacol..

[75] Rabizadeh S., Nakhjavani M., Esteghamati A. (2019). Cardiovascular and Renal Benefits of SGLT2 Inhibitors: A Narrative Review. Int. J. Endocrinol. Metab..

[76] Vlotides G., Mertens P.R. (2014). Sodium-glucose cotransport inhibitors: Mechanisms, metabolic effects and implications for the treatment of diabetic patients with chronic kidney disease: FIGURE 1. Nephrol. Dial. Transplant..

[77] Vallon V., Rose M., Gerasimova M., Satriano J., Platt K.A., Koepsell H., Cunard R., Sharma K., Thomson S.C., Rieg T. (2013). Knockout of Na-glucose transporter SGLT2 attenuates hyperglycemia and glomerular hyperfiltration but not kidney growth or injury in diabetes mellitus. Am. J. Physiol. Physiol..

[78] Vallon V., Schroth J., Satriano J., Blantz R.C., Thomson S.C., Rieg T. (2009). Adenosine A1 Receptors Determine Glomerular Hyperfiltration and the Salt Paradox in Early Streptozotocin Diabetes Mellitus. Nephron Physiol..

[79] Vallon V., Thomson S.C. (2012). Renal Function in Diabetic Disease Models: The Tubular System in the Pathophysiology of the Diabetic Kidney. Annu. Rev. Physiol..

[80] Tonneijck L., Muskiet M., Smits M., Van Bommel E.J., Heerspink H.J.L., Van Raalte D.H., Joles J.A. (2017). Glomerular Hyperfiltration in Diabetes: Mechanisms, Clinical Significance, and Treatment. J. Am. Soc. Nephrol..

[81] Evans R.G., Harrop G.K., Ngo J., Ow P.C.C., O’Connor P.M. (2014). Basal renal O2 consumption and the efficiency of O2 utilization for Na^+^ reabsorption. Am. J. Physiol. Physiol..

[82] Takiyama Y., Haneda M. (2014). Hypoxia in Diabetic Kidneys. BioMed Res. Int..

[83] Layton A.T., Laghmani K., Vallon V., Edwards A. (2016). Solute transport and oxygen consumption along the nephrons: Effects of Na^+^ transport inhibitors. Am. J. Physiol. Physiol..

[84] Persson P., Palm F. (2017). Hypoxia-inducible factor activation in diabetic kidney disease. Curr. Opin. Nephrol. Hypertens..

[85] García-Pastor C., Benito-Martínez S., Moreno-Manzano V., Fernández-Martínez A.B., De Lucio-Cazaña F.J. (2019). Mechanism and Consequences of The Impaired Hif-1α Response to Hypoxia in Human Proximal Tubular HK-2 Cells Exposed to High Glucose. Sci. Rep..

[86] Basile D.P., Donohoe D., Roethe K., Osborn J.L. (2001). Renal ischemic injury results in permanent damage to peritubular capillaries and influences long-term function. Am. J. Physiol. Physiol..

[87] Masarone M., Rosato V., Aglitti A., Bucci T., Caruso R., Salvatore T., Sasso F.C., Tripodi M.F., Persico M. (2017). Liver biopsy in type 2 diabetes mellitus: Steatohepatitis represents the sole feature of liver damage. PLoS ONE.

[88] Sasso F.C., Salvatore T., Tranchino G., Cozzolino D., Caruso A.A., Persico M., Gentile S., Torella D., Torella R. (1999). Cochlear dysfunction in type 2 diabetes: A complication independent of neuropathy and acute hyperglycemia. Metabolism.

[89] Rizzo M.R., Sasso F.C., Marfella R., Siniscalchi M., Paolisso P., Carbonara O., Capoluongo M.C., Lascar N., Pace C., Sardu C. (2015). Autonomic dysfunction is associated with brief episodes of atrial fibrillation in type 2 diabetes. J. Diabetes Complicat..

[90] Galiero R., Ricciardi D., Pafundi P.C., Todisco V., Tedeschi G., Cirillo G., Sasso F.C. (2021). Whole plantar nerve conduction study: A new tool for early diagnosis of peripheral diabetic neuropathy. Diabetes Res. Clin. Pract..

[91] Sasso F.C., Pafundi P.C., Gelso A., Bono V., Costagliola C., Marfella R., Sardu C., Rinaldi L., Galiero R., Acierno C. (2018). Applicability of telemedicine in the screening of diabetic retinopathy (DR): The first multicentre study in Italy. The No Blind Study. Diabetes/Metab. Res. Rev..

[92] Cannon C.P., Pratley R., Dagogo-Jack S., Mancuso J., Huyck S., Masiukiewicz U., Charbonnel B., Frederich R., Gallo S., Cosentino F. (2020). Cardiovascular Outcomes with Ertugliflozin in Type 2 Diabetes. N. Engl. J. Med..

[93] Bhatt D.L., Szarek M., Pitt B., Cannon C.P., Leiter L.A., McGuire D.K., Lewis J.B., Riddle M.C., Inzucchi S.E., Kosiborod M.N. (2021). Sotagliflozin in Patients with Diabetes and Chronic Kidney Disease. N. Engl. J. Med..

[94] Zelniker T.A., Wiviott S.D., Raz I., Im K., Goodrich E., Bonaca M.P., Mosenzon O., Kato E., Cahn A., Furtado R.H.M. (2019). SGLT2 inhibitors for primary and secondary prevention of cardiovascular and renal outcomes in type 2 diabetes: A systematic review and meta-analysis of cardiovascular outcome trials. Lancet.

[95] Cosentino F., Grant P.J., Aboyans V., Bailey C.J., Ceriello A., Delgado V., Federici M., Filippatos G., Grobbee D.E., Hansen T.B. (2020). 2019 ESC Guidelines on diabetes, pre-diabetes, and cardiovascular diseases developed in collaboration with the EASD. Eur. Heart J..

[96] Verma S., McMurray J.J.V., Cherney D.Z.I. (2017). The Metabolodiuretic Promise of Sodium-Dependent Glucose Cotransporter 2 Inhibition. JAMA Cardiol..

[97] Sasso F.C., Chiodini P., Carbonara O., De Nicola L., Conte G., Salvatore T., Nasti R., Marfella R., Gallo C., Signoriello S. (2011). High cardiovascular risk in patients with Type 2 diabetic nephropathy: The predictive role of albuminuria and glomerular filtration rate. The NID-2 Prospective Cohort Study. Nephrol. Dial. Transplant..

[98] Sasso F.C., De Nicola L., Carbonara O., Nasti R., Minutolo R., Salvatore T., Conte G., Torella R. (2006). Cardiovascular Risk Factors and Disease Management in Type 2 Diabetic Patients With Diabetic Nephropathy. Diabetes Care.

[99] Minutolo R., Sasso F.C., Chiodini P., Cianciaruso B., Carbonara O., Zamboli P., Tirino G., Pota A., Torella R., Conte G. (2006). Management of cardiovascular risk factors in advanced type 2 diabetic nephropathy: A comparative analysis in nephrology, diabetology and primary care settings. J. Hypertens..

[100] McMurray J.J.V., Solomon S.D., Inzucchi S.E., Køber L., Kosiborod M.N., Martinez F.A., Ponikowski P., Sabatine M.S., Anand I.S., Bělohlávek J. (2019). Dapagliflozin in Patients with Heart Failure and Reduced Ejection Fraction. N. Engl. J. Med..

[101] Packer M., Anker S.D., Butler J., Filippatos G., Pocock S.J., Carson P., Januzzi J., Verma S., Tsutsui H., Brueckmann M. (2020). Cardiovascular and Renal Outcomes with Empagliflozin in Heart Failure. N. Engl. J. Med..

[102] Bhatt D.L., Szarek M., Steg P.G., Cannon C.P., Leiter L.A., McGuire D.K., Lewis J.B., Riddle M.C., Voors A.A., Metra M. (2021). Sotagliflozin in Patients with Diabetes and Recent Worsening Heart Failure. N. Engl. J. Med..

[103] Nassif M.E., Windsor S.L., Tang F., Khariton Y., Husain M., Inzucchi S.E., Mc-Guire D.K., Pitt B., Scirica B.M., Austin B. (2019). Dapagliflozin Effects on Biomarkers, Symptoms, and Functional Status in Patients With Heart Failure With Reduced Ejection Fraction. Circulation.

[104] Abraham W.T., Lindenfeld J., Ponikowski P., Agostoni P., Butler J., Desai A.S., Filippatos G., Gniot J., Fu M., Gullestad L. (2020). Effect of empagliflozin on exercise ability and symptoms in heart failure patients with reduced and preserved ejection fraction, with and without type 2 diabetes. Eur. Heart J..

[105] Perkovic V., Jardine M.J., Neal B., Bompoint S., Heerspink H.J.L., Charytan D.M., Edwards R., Agarwal R., Bakris G., Bull S. (2019). Canagliflozin and Renal Outcomes in Type 2 Diabetes and Nephropathy. N. Engl. J. Med..

[106] Heerspink H.J.L., Stefánsson B.V., Correa-Rotter R., Chertow G.M., Greene T., Hou F.-F., Mann J.F.E., McMurray J.J.V., Lindberg M., Rossing P. (2020). Dapagliflozin in Patients with Chronic Kidney Disease. N. Engl. J. Med..

[107] Pollock C., Stefánsson B., Reyner D., Rossing P., Sjöström C.D., Wheeler D.C., Langkilde A.M., Heerspink H.J.L. (2019). Albuminuria-lowering effect of dapagliflozin alone and in combination with saxagliptin and effect of dapagliflozin and saxagliptin on glycaemic control in patients with type 2 diabetes and chronic kidney disease (DELIGHT): A randomised, double-blind, placebo-controlled trial. Lancet Diabetes Endocrinol..

[108] Fioretto P., Del Prato S., Buse J.B., Goldenberg R., Giorgino F., Reyner D., Langkilde A.M., Sjöström C.D., Sartipy P. (2018). On Behalf of the DERIVE Study Investigators Efficacy and safety of dapagliflozin in patients with type 2 diabetes and moderate renal impairment (chronic kidney disease stage 3A): The DERIVE Study. Diabetes, Obes. Metab..

[109] Cherney D.Z.I., Dekkers C.C.J., Barbour S.J., Cattran D., Gafor A.H.A., Greasley P.J., Laverman G.D., Lim S.K., Di Tanna G.L., Reich H.N. (2020). Effects of the SGLT2 inhibitor dapagliflozin on proteinuria in non-diabetic patients with chronic kidney disease (DIAMOND): A randomised, double-blind, crossover trial. Lancet Diabetes Endocrinol..

[110] McDonagh T.A., Metra M., Adamo M., Gardner R.S., Baumbach A., Böhm M., Burri H., Butler J., Čelutkienė J., Chioncel O. (2021). 2021 ESC Guidelines for the diagnosis and treatment of acute and chronic heart failure: Developed by the Task Force for the Diagnosis and Treatment of Acute and Chronic Heart Failure of the European Society of Cardiology (ESC) With the Special Contribution of the Heart Failure Association (HFA) of the ESC. Eur. Heart J..

[111] Yancy C.W., Januzzi J.L., Allen L.A., Butler J., Davis L.L., Fonarow G.C., Ibrahim N.E., Jessup M., Lindenfeld J., Maddox T.M. (2018). 2017 ACC Expert Consensus Decision Pathway for Optimization of Heart Failure Treatment: Answers to 10 Pivotal Issues About Heart Failure With Reduced Ejection Fraction. J. Am. Coll. Cardiol..

[112] Draznin B., Aroda V.R., Bakris G., Benson G., Brown F.M., Freeman R., Green J., Huang E., American Diabetes Association Professional Practice, Committee, American Diabetes Association Professional Practice, Committee (2022). Cardiovascular Disease and Risk Management: Standards of Medical Care in Diabetes-2022. Diabetes Care.

[113] Draznin B., Aroda V.R., Bakris G., Benson G., Brown F.M., Freeman R., Green J., Huang E., American Diabetes Association Professional Practice, Committee, American Diabetes Association Professional Practice, Committee (2022). Chronic Kidney Disease and Risk Management: Standards of Medical Care in Diabetes-2022. Diabetes Care.

[114] Anker S.D., Butler J., Filippatos G., Ferreira J.P., Bocchi E., Böhm M., Brunner–La Rocca H.-P., Choi D.-J., Chopra V., Chuquiure-Valenzuela E. (2021). Empagliflozin in Heart Failure with a Preserved Ejection Fraction. N. Engl. J. Med..

[115] Packer M., Butler J., Zannad F., Filippatos G., Ferreira J.P., Pocock S.J., Carson P., Anand I., Doehner W., Haass M. (2021). Effect of Empagliflozin on Worsening Heart Failure Events in Patients With Heart Failure and Preserved Ejection Fraction: EMPEROR-Preserved Trial. Circulation.

[116] Anker S.D., Butler J., Filippatos G., Khan M.S., Ferreira J.P., Bocchi E., Böhm M., Rocca H.P.B., Choi D., Chopra V. (2020). Baseline characteristics of patients with heart failure with preserved ejection fraction in the EMPEROR-Preserved trial. Eur. J. Heart Fail..

[117] Brenner B.M. (1983). Hemodynamically mediated glomerular injury and the progressive nature of kidney disease. Kidney Int..

[118] Thomson S.C., Rieg T., Miracle C., Mansoury H., Whaley J., Vallon V., Singh P. (2012). Acute and chronic effects of SGLT2 blockade on glomerular and tubular function in the early diabetic rat. Am. J. Physiol. Integr. Comp. Physiol..

[119] Terami N., Ogawa D., Tachibana H., Hatanaka T., Wada J., Nakatsuka A., Eguchi J., Horiguchi C.S., Nishii N., Yamada H. (2014). Long-Term Treatment with the Sodium Glucose Cotransporter 2 Inhibitor, Dapagliflozin, Ameliorates Glucose Homeostasis and Diabetic Nephropathy in db/db Mice. PLoS ONE.

[120] Cherney D.Z., Perkins B.A., Soleymanlou N., Maione M., Lai V., Lee A., Fagan N.M., Woerle H.J., Johansen O.E., Broedl U.C. (2014). Renal Hemodynamic Effect of Sodium-Glucose Cotransporter 2 Inhibition in Patients With Type 1 Diabetes Mellitus. Circulation.

[121] Škrtić M., Yang G., Perkins B.A., Soleymanlou N., Lytvyn Y., Von Eynatten M., Woerle H.J., Johansen O.E., Broedl U.C., Hach T. (2014). Characterisation of glomerular haemodynamic responses to SGLT2 inhibition in patients with type 1 diabetes and renal hyperfiltration. Diabetologia.

[122] Kidokoro K., Cherney D.Z.I., Bozovic A., Nagasu H., Satoh M., Kanda E., Sasaki T., Kashihara N. (2019). Evaluation of Glomerular Hemodynamic Function by Empagliflozin in Diabetic Mice Using In Vivo Imaging. Circulation.

[123] van Bommel E.J., Muskiet M., van Baar M.J., Tonneijck L., Smits M., Emanuel A.L., Bozovic A., Danser A.J., Geurts F., Hoorn E.J. (2020). The renal hemodynamic effects of the SGLT2 inhibitor dapagliflozin are caused by post-glomerular vasodilatation rather than pre-glomerular vasoconstriction in metformin-treated patients with type 2 diabetes in the randomized, double-blind RED trial. Kidney Int..

[124] Heerspink H.J.L., Perkins B.A., Fitchett D.H., Husain M., Cherney D.Z.I. (2016). Sodium Glucose Cotransporter 2 Inhibitors in the Treatment of Diabetes Mellitus. Circulation.

[125] Wanner C., Heerspink H.J., Zinman B., Inzucchi S.E., Koitka-Weber A., Mattheus M., Hantel S., Woerle H.-J., Broedl U.C., von Eynatten M. (2018). Empagliflozin and Kidney Function Decline in Patients with Type 2 Diabetes: A Slope Analysis from the EMPA-REG OUTCOME Trial. J. Am. Soc. Nephrol..

[126] De Nicola L., Gabbai F.B., Garofalo C., Conte G., Minutolo R. (2020). Nephroprotection by SGLT2 Inhibition: Back to the Future?. J. Clin. Med..

[127] Heerspink H.J., Kosiborod M., Inzucchi S.E., Cherney D.Z. (2018). Renoprotective effects of sodium-glucose cotransporter-2 inhibitors. Kidney Int..

[128] Cherney D.Z.I., Zinman B., Inzucchi S.E., Koitka-Weber A., Mattheus M., von Eynatten M., Wanner C. (2017). Effects of empagliflozin on the urinary albumin-to-creatinine ratio in patients with type 2 diabetes and established cardiovascular disease: An exploratory analysis from the EMPA-REG OUTCOME randomised, placebo-controlled trial. Lancet Diabetes Endocrinol..

[129] Wanner C., Inzucchi S.E., Lachin J.M., Fitchett D., Von Eynatten M., Mattheus M., Johansen O.E., Woerle H.J., Broedl U.C., Zinman B. (2016). Empagliflozin and Progression of Kidney Disease in Type 2 Diabetes. N. Engl. J. Med..

[130] Giugliano D., De Nicola L., Maiorino M.I., Bellastella G., Garofalo C., Chiodini P., Ceriello A., Esposito K. (2020). Preventing major adverse cardiovascular events by SGLT-2 inhibition in patients with type 2 diabetes: The role of kidney. Cardiovasc. Diabetol..

[131] Steffes M.W., Schmidt D., Mccrery R., Basgen J.M., International Diabetic Nephropathy Study Group (2001). Glomerular cell number in normal subjects and in type 1 diabetic patients. Kidney Int..

[132] Pagtalunan M.E., Miller P.L., Jumping-Eagle S., Nelson R.G., Myers B.D., Rennke H.G., Coplon N.S., Sun L., Meyer T.W. (1997). Podocyte loss and progressive glomerular injury in type II diabetes. J. Clin. Investig..

[133] Cassis P., Locatelli M., Cerullo D., Corna D., Buelli S., Zanchi C., Villa S., Morigi M., Remuzzi G., Benigni A. (2018). SGLT2 inhibitor dapagliflozin limits podocyte damage in proteinuric nondiabetic nephropathy. JCI Insight.

[134] Tanaka S., Tanaka T., Nangaku M. (2014). Hypoxia as a key player in the AKI-to-CKD transition. Am. J. Physiol. Physiol..

[135] Hesp A.C., Schaub J.A., Prasad P.V., Vallon V., Laverman G.D., Bjornstad P., van Raalte D.H. (2020). The role of renal hypoxia in the pathogenesis of diabetic kidney disease: A promising target for newer renoprotective agents including SGLT2 inhibitors?. Kidney Int..

[136] Ganz M.B., Hawkins K., Reilly R.F. (2000). High glucose induces the activity and expression of Na(+)/H(+) exchange in glomerular mesangial cells. Am. J. Physiol. Physiol..

[137] Chen J., Williams S., Ho S., Loraine H., Hagan D., Whaley J.M., Feder J.N. (2010). Quantitative PCR tissue expression profiling of the human SGLT2 gene and related family members. Diabetes Ther..

[138] Chen-Izu Y., Shaw R.M., Pitt G.S., Yarov-Yarovoy V., Sack J.T., Abriel H., Aldrich R.W., Belardinelli L., Cannell M.B., Catterall W.A. (2015). Na^+^ channel function, regulation, structure, trafficking and sequestration. J. Physiol..

[139] Trum M., Riechel J., Wagner S. (2021). Cardioprotection by SGLT2 Inhibitors—Does It All Come Down to Na^+^?. Int. J. Mol. Sci..

[140] Bertero E., Prates Roma L., Ameri P., Maack C. (2018). Cardiac effects of SGLT2 inhibitors: The sodium hypothesis. Cardiovasc. Res..

[141] Mustroph J., Neef S., Maier L.S. (2017). CaMKII as a target for arrhythmia suppression. Pharmacol. Ther..

[142] Mustroph J., Sag C.M., Bähr F., Schmidtmann A.-L., Gupta S.N., Dietz A., Islam M.T., Lücht C.M., Beuthner B.E., Pabel S. (2021). Loss of CASK Accelerates Heart Failure Development. Circ. Res..

[143] Pabel S., Hamdani N., Luedde M., Sossalla S. (2021). SGLT2 Inhibitors and Their Mode of Action in Heart Failure—Has the Mystery Been Unravelled?. Curr. Heart Fail. Rep..

[144] Yu S., Li G., Huang C.L.-H., Lei M., Wu L. (2017). Late sodium current associated cardiac electrophysiological and mechanical dysfunction. Pflügers Arch.-Eur. J. Physiol..

[145] Baartscheer A., Schumacher C.A., Van Borren M.M.G.J., Belterman C.N.W., Coronel R., Fiolet J.W.T. (2003). Increased Na^+^/H^+^-exchange activity is the cause of increased [Na^+^]i and underlies disturbed calcium handling in the rabbit pressure and volume overload heart failure model. Cardiovasc. Res..

[146] Packer M. (2017). Activation and Inhibition of Sodium-Hydrogen Exchanger Is a Mechanism That Links the Pathophysiology and Treatment of Diabetes Mellitus With That of Heart Failure. Circulation.

[147] Studer R., Reinecke H., Bilger J., Eschenhagen T., Böhm M., Hasenfuss G., Just H., Holtz J., Drexler H. (1994). Gene expression of the cardiac Na(^+^)-Ca^2+^ exchanger in end-stage human heart failure. Circ. Res..

[148] Sayour A.A., Oláh A., Ruppert M., Barta B.A., Horváth E.M., Benke K., Pólos M., Hartyánszky I., Merkely B., Radovits T. (2020). Characterization of left ventricular myocardial sodium-glucose cotransporter 1 expression in patients with end-stage heart failure. Cardiovasc. Diabetol..

[149] Baartscheer A., Schumacher C.A., Wust R.C., Fiolet J.W.T., Stienen G., Coronel R., Zuurbier C.J. (2016). Empagliflozin decreases myocardial cytoplasmic Na^+^ through inhibition of the cardiac Na^+^/H^+^ exchanger in rats and rabbits. Diabetologia.

[150] Uthman L., Baartscheer A., Bleijlevens B., Schumacher C.A., Fiolet J.W.T., Koeman A., Jancev M., Hollmann M.W., Weber N.C., Coronel R. (2017). Class effects of SGLT2 inhibitors in mouse cardiomyocytes and hearts: Inhibition of Na^+^/H^+^ exchanger, lowering of cytosolic Na^+^ and vasodilation. Diabetologia.

[151] Trum M., Riechel J., Lebek S., Pabel S., Sossalla S.T., Hirt S., Arzt M., Maier L.S., Wagner S. (2020). Empagliflozin inhibits Na^+^ /H^+^ exchanger activity in human atrial cardiomyocytes. ESC Heart Fail..

[152] Philippaert K., Kalyaanamoorthy S., Fatehi M., Long W., Soni S., Byrne N.J., Barr A., Singh J., Wong J., Palechuk T. (2021). Cardiac Late Sodium Channel Current Is a Molecular Target for the Sodium/Glucose Cotransporter 2 Inhibitor Empagliflozin. Circulation.

[153] Lapuerta P., Zambrowicz B., Strumph P., Sands A. (2015). Development of sotagliflozin, a dual sodium-dependent glucose transporter 1/2 inhibitor. Diabetes Vasc. Dis. Res..

[154] Mustroph J., Wagemann O., Lücht C.M., Trum M., Hammer K., Sag C.M., Lebek S., Tarnowski D., Reinders J., Perbellini F. (2018). Empagliflozin reduces Ca/calmodulin-dependent kinase II activity in isolated ventricular cardiomyocytes. ESC Heart Fail..

[155] Maejima Y. (2020). SGLT2 Inhibitors Play a Salutary Role in Heart Failure via Modulation of the Mitochondrial Function. Front. Cardiovasc. Med..

[156] Sasso F.C., Pafundi P.C., Simeon V., De Nicola L., Chiodini P., Galiero R., Rinaldi L., Nevola R., Salvatore T., Sardu C. (2021). Efficacy and durability of multifactorial intervention on mortality and MACEs: A randomized clinical trial in type-2 diabetic kidney disease. Cardiovasc. Diabetol..

[157] Abdul-Ghani M., Del Prato S., Chilton R., DeFronzo R.A. (2016). SGLT2 Inhibitors and Cardiovascular Risk: Lessons Learned From the EMPA-REG OUTCOME Study. Diabetes Care.

[158] Butler J., Hamo C.E., Filippatos G., Pocock S.J., Bernstein R.A., Brueckmann M., Cheung A.K., George J.T., Green J.B., Januzzi J.L. (2017). The potential role and rationale for treatment of heart failure with sodium-glucose co-transporter 2 inhibitors. Eur. J. Heart Fail..

[159] Inzucchi S.E., Zinman B., Fitchett D., Wanner C., Ferrannini E., Schumacher M., Schmoor C., Ohneberg K., Johansen O.E., George J.T. (2017). How Does Empagliflozin Reduce Cardiovascular Mortality? Insights From a Mediation Analysis of the EMPA-REG OUTCOME Trial. Diabetes Care.

[160] Koye D.N., Magliano D., Nelson R.G., Pavkov M.E. (2018). The Global Epidemiology of Diabetes and Kidney Disease. Adv. Chronic Kidney Dis..

[161] Maeda S., Matsui T., Takeuchi M., Yamagishi S.-I. (2013). Sodium-glucose cotransporter 2-mediated oxidative stress augments advanced glycation end products-induced tubular cell apoptosis. Diabetes Metab. Res. Rev..

[162] Ferrannini G., Hach T., Crowe S., Sanghvi A., Hall K.D., Ferrannini E. (2015). Energy Balance After Sodium–Glucose Cotransporter 2 Inhibition. Diabetes Care.

[163] Sa-Nguanmoo P., Tanajak P., Kerdphoo S., Jaiwongkam T., Pratchayasakul W., Chattipakorn N., Chattipakorn S.C. (2017). SGLT2-inhibitor and DPP-4 inhibitor improve brain function via attenuating mitochondrial dysfunction, insulin resistance, inflammation, and apoptosis in HFD-induced obese rats. Toxicol. Appl. Pharmacol..

[164] Millar P., Pathak N., Parthsarathy V., Bjourson A.J., O’Kane M., Pathak V., Moffett R.C., Flatt P.R., Gault V.A. (2017). Metabolic and neuroprotective effects of dapagliflozin and liraglutide in diabetic mice. J. Endocrinol..

[165] Wang H., Yang J., Chen X., Qiu F., Li J. (2019). Effects of Sodium-glucose Cotransporter 2 Inhibitor Monotherapy on Weight Changes in Patients With Type 2 Diabetes Mellitus: A Bayesian Network Meta-analysis. Clin. Ther..

[166] Bolinder J., Ljunggren Ö., Kullberg J., Johansson L., Wilding J., Langkilde A.M., Sugg J., Parikh S. (2012). Effects of Dapagliflozin on Body Weight, Total Fat Mass, and Regional Adipose Tissue Distribution in Patients with Type 2 Diabetes Mellitus with Inadequate Glycemic Control on Metformin. J. Clin. Endocrinol. Metab..

[167] Brown E., Wilding J.P., Barber T.M., Alam U., Cuthbertson D.J. (2019). Weight loss variability with SGLT2 inhibitors and GLP-1 receptor agonists in type 2 diabetes mellitus and obesity: Mechanistic possibilities. Obes. Rev..

[168] Zheng R., Zhou D., Zhu Y. (2016). The long-term prognosis of cardiovascular disease and all-cause mortality for metabolically healthy obesity: A systematic review and meta-analysis. J. Epidemiol. Community Health.

[169] Foster M.C., Hwang S.-J., Larson M., Lichtman J.H., Parikh N.I., Vasan R.S., Levy D., Fox C.S. (2008). Overweight, Obesity, and the Development of Stage 3 CKD: The Framingham Heart Study. Am. J. Kidney Dis..

[170] Hsu C.-Y., McCulloch C.E., Iribarren C., Darbinian J., Go A.S. (2006). Body Mass Index and Risk for End-Stage Renal Disease. Ann. Intern. Med..

[171] Tamura K., Wakui H., Azushima K., Uneda K., Umemura S. (2016). Circadian blood pressure rhythm as a possible key target of SGLT2 inhibitors used for the treatment of Type 2 diabetes. Hypertens. Res..

[172] Rahman A., Hitomi H., Nishiyama A. (2017). Cardioprotective effects of SGLT2 inhibitors are possibly associated with normalization of the circadian rhythm of blood pressure. Hypertens. Res..

[173] Georgianos P.I., Agarwal R. (2019). Ambulatory Blood Pressure Reduction With SGLT-2 Inhibitors: Dose-Response Meta-analysis and Comparative Evaluation With Low-Dose Hydrochlorothiazide. Diabetes Care.

[174] Majewski C., Bakris G.L. (2015). Blood Pressure Reduction: An Added Benefit of Sodium–Glucose Cotransporter 2 Inhibitors in Patients With Type 2 Diabetes. Diabetes Care.

[175] Puglisi S., Rossini A., Poli R., Dughera F., Pia A., Terzolo M., Reimondo G. (2021). Effects of SGLT2 Inhibitors and GLP-1 Receptor Agonists on Renin-Angiotensin-Aldosterone System. Front. Endocrinol..

[176] Cefalu W.T., Stenlöf K., Leiter L.A., Wilding J., Blonde L., Polidori D., Xie J., Sullivan D., Usiskin K., Canovatchel W. (2015). Effects of canagliflozin on body weight and relationship to HbA1c and blood pressure changes in patients with type 2 diabetes. Diabetologia.

[177] Sjöström C.D., Johansson P., Ptaszynska A., List J., Johnsson E. (2015). Dapagliflozin lowers blood pressure in hypertensive and non-hypertensive patients with type 2 diabetes. Diabetes Vasc. Dis. Res..

[178] Dimova R., Tankova T. (2020). Does SGLT2 Inhibition Affect Sympathetic Nerve Activity in Type 2 Diabetes?. Horm. Metab. Res..

[179] Sugiyama S., Jinnouchi H., Kurinami N., Hieshima K., Yoshida A., Jinnouchi K., Nishimura H., Suzuki T., Miyamoto F., Kajiwara K. (2018). The SGLT2 Inhibitor Dapagliflozin Significantly Improves the Peripheral Microvascular Endothelial Function in Patients with Uncontrolled Type 2 Diabetes Mellitus. Intern. Med..

[180] Chilton R., Tikkanen I., Cannon C.P., Crowe S., Woerle H.J., Broedl U.C., Johansen O.E. (2015). Effects of empagliflozin on blood pressure and markers of arterial stiffness and vascular resistance in patients with type 2 diabetes. Diabetes Obes. Metab..

[181] Benham J.L., Booth J.E., Sigal R.J., Daskalopoulou S.S., Leung A.A., Rabi D.M. (2021). Systematic review and meta-analysis: SGLT2 inhibitors, blood pressure and cardiovascular outcomes. Int. J. Cardiol. Heart Vasc..

[182] Guo M., Ding J., Li J., Wang J., Zhang T., Liu C., Huang W., Long Y., Gao C., Xu Y. (2018). SGLT2 inhibitors and risk of stroke in patients with type 2 diabetes: A systematic review and meta-analysis. Diabetes Obes. Metab..

[183] Zhou Z., Jardine M.J., Li Q., Neuen B.L., Cannon C.P., de Zeeuw D., Edwards R., Levin A., Mahaffey K.W., Perkovic V. (2021). Effect of SGLT2 Inhibitors on Stroke and Atrial Fibrillation in Diabetic Kidney Disease. Stroke.

[184] Scheen A., Delanaye P. (2017). Effects of reducing blood pressure on renal outcomes in patients with type 2 diabetes: Focus on SGLT2 inhibitors and EMPA-REG OUTCOME. Diabetes Metab..

[185] Wang J., Chen Y., Xu W., Lu N., Cao J., Yu S. (2019). Effects of intensive blood pressure lowering on mortality and cardiovascular and renal outcomes in type 2 diabetic patients: A meta-analysis. PLoS ONE.

[186] Kimura Y., Tsukui D., Kono H. (2021). Uric Acid in Inflammation and the Pathogenesis of Atherosclerosis. Int. J. Mol. Sci..

[187] Goldberg A., Garcia-Arroyo F., Sasai F., Rodriguez-Iturbe B., Sanchez-Lozada L.G., Lanaspa M.A., Johnson R.J. (2021). Mini Review: Reappraisal of Uric Acid in Chronic Kidney Disease. Am. J. Nephrol..

[188] Zhao Y., Xu L., Tian D., Xia P., Zheng H., Wang L., Chen L. (2017). Effects of sodium-glucose co-transporter 2 (SGLT2) inhibitors on serum uric acid level: A meta-analysis of randomized controlled trials. Diabetes Obes. Metab..

[189] Novikov A., Fu Y., Huang W., Freeman B., Patel R., Van Ginkel C., Koepsell H., Busslinger M., Onishi A., Nespoux J. (2019). SGLT2 inhibition and renal urate excretion: Role of luminal glucose, GLUT9, and URAT1. Am. J. Physiol. Physiol..

[190] Calapkulu M., Cander S., Gul O.O., Ersoy C. (2019). Lipid profile in type 2 diabetic patients with new dapagliflozin treatment; actual clinical experience data of six months retrospective lipid profile from single center. Diabetes Metab. Syndr. Clin. Res. Rev..

[191] Inagaki N., Goda M., Yokota S., Maruyama N., Iijima H. (2015). Effects of Baseline Blood Pressure and Low-Density Lipoprotein Cholesterol on Safety and Efficacy of Canagliflozin in Japanese Patients with Type 2 Diabetes Mellitus. Adv. Ther..

[192] Halimi S., Vergès B. (2014). Adverse effects and safety of SGLT-2 inhibitors. Diabetes Metab..

[193] Szekeres Z., Toth K., Szabados E. (2021). The Effects of SGLT2 Inhibitors on Lipid Metabolism. Metabolites.

[194] Hayashi T., Fukui T., Nakanishi N., Yamamoto S., Tomoyasu M., Osamura A., Ohara M., Yamamoto T., Ito Y., Hirano T. (2017). Dapagliflozin decreases small dense low-density lipoprotein-cholesterol and increases high-density lipoprotein 2-cholesterol in patients with type 2 diabetes: Comparison with sitagliptin. Cardiovasc. Diabetol..

[195] Kamijo Y., Ishii H., Yamamoto T., Kobayashi K., Asano H., Miake S., Kanda E., Urata H., Yoshida M. (2019). Potential Impact on Lipoprotein Subfractions in Type 2 Diabetes. Clin. Med. Insights: Endocrinol. Diabetes.

[196] Liu Y., Xu J., Wu M., Xu B., Kang L. (2021). Empagliflozin protects against atherosclerosis progression by modulating lipid profiles and sympathetic activity. Lipids Health Dis..

[197] Chen M.-B., Wang H., Cui W.-Y., Xu H.-L., Zheng Q.-H. (2021). Effect of SGLT inhibitors on weight and lipid metabolism at 24 weeks of treatment in patients with diabetes mellitus. Medicine.

[198] Filippas-Ntekouan S., Tsimihodimos V., Filippatos T., Dimitriou T., Elisaf M. (2018). SGLT-2 inhibitors: Pharmacokinetics characteristics and effects on lipids. Expert Opin. Drug Metab. Toxicol..

[199] Lazarte J., Kanagalingam T., Hegele R.A. (2021). Lipid effects of sodium-glucose cotransporter 2 inhibitors. Curr. Opin. Lipidol..

[200] Liu Z., Ma X., Ilyas I., Zheng X., Luo S., Little P.J., Kamato D., Sahebkar A., Wu W., Weng J. (2021). Impact of sodium glucose cotransporter 2 (SGLT2) inhibitors on atherosclerosis: From pharmacology to pre-clinical and clinical therapeutics. Theranostics.

[201] Barraclough J.Y., Patel S., Yu J., Neal B., Arnott C. (2021). The Role of Sodium Glucose Cotransporter-2 Inhibitors in Atherosclerotic Cardiovascular Disease: A Narrative Review of Potential Mechanisms. Cells.

[202] Daniele G., Xiong J., Solis-Herrera C., Merovci A., Eldor R., Tripathy D., DeFronzo R.A., Norton L., Abdul-Ghani M. (2016). Dapagliflozin Enhances Fat Oxidation and Ketone Production in Patients With Type 2 Diabetes. Diabetes Care.

[203] Ferrannini E., Baldi S., Frascerra S., Astiarraga B., Heise T., Bizzotto R., Mari A., Pieber T.R., Muscelli E. (2016). Shift to Fatty Substrate Utilization in Response to Sodium–Glucose Cotransporter 2 Inhibition in Subjects Without Diabetes and Patients With Type 2 Diabetes. Diabetes.

[204] Ferrannini E., Baldi S., Frascerra S., Astiarraga B., Barsotti E., Clerico A., Muscelli E. (2017). Renal Handling of Ketones in Response to Sodium–Glucose Cotransporter 2 Inhibition in Patients With Type 2 Diabetes. Diabetes Care.

[205] Osataphan S., Macchi C., Singhal G., Chimene-Weiss J., Sales V., Kozuka C., Dreyfuss J., Pan H., Tangcharoenpaisan Y., Morningstar J. (2019). SGLT2 inhibition reprograms systemic metabolism via FGF21-dependent and -independent mechanisms. JCI Insight.

[206] Tezze C., Romanello V., Sandri M. (2019). FGF21 as Modulator of Metabolism in Health and Disease. Front. Physiol..

[207] Avogaro A., Fadini G.P., Del Prato S. (2020). Reinterpreting Cardiorenal Protection of Renal Sodium–Glucose Cotransporter 2 Inhibitors via Cellular Life History Programming. Diabetes Care.

[208] Aubert G., Martin O.J., Horton J.L., Lai L., Vega R.B., Leone T.C., Koves T., Gardell S.J., Krüger M., Hoppel C.L. (2016). The Failing Heart Relies on Ketone Bodies as a Fuel. Circulation.

[209] Dutka M., Bobiński R., Ulman-Włodarz I., Hajduga M., Bujok J., Pająk C., Ćwiertnia M. (2020). Sodium glucose cotransporter 2 inhibitors: Mechanisms of action in heart failure. Heart Fail. Rev..

[210] Hattori Y. (2021). Beneficial effects on kidney during treatment with sodium-glucose cotransporter 2 inhibitors: Proposed role of ketone utilization. Heart Fail. Rev..

[211] Bedi K.C., Snyder N.W., Brandimarto J., Aziz M., Mesaros C., Worth A.J., Wang L.L., Javaheri A., Blair I.A., Margulies K.B. (2016). Evidence for Intramyocardial Disruption of Lipid Metabolism and Increased Myocardial Ketone Utilization in Advanced Human Heart Failure. Circulation.

[212] Sun H., Olson K.C., Gao C., Prosdocimo D.A., Zhou M., Wang Z., Jeyaraj D., Youn J.-Y., Ren S., Liu Y. (2016). Catabolic Defect of Branched-Chain Amino Acids Promotes Heart Failure. Circulation.

[213] Kappel B.A., Lehrke M., Schütt K., Artati A., Adamski J., Lebherz C., Marx N. (2017). Effect of Empagliflozin on the Metabolic Signature of Patients With Type 2 Diabetes Mellitus and Cardiovascular Disease. Circulation.

[214] Ferrannini E., Mark M., Mayoux E. (2016). CV Protection in the EMPA-REG OUTCOME Trial: A “Thrifty Substrate” Hypothesis. Diabetes Care.

[215] Mudaliar S., Alloju S., Henry R.R. (2016). Can a Shift in Fuel Energetics Explain the Beneficial Cardiorenal Outcomes in the EMPA-REG OUTCOME Study? A Unifying Hypothesis. Diabetes Care.

[216] Verma S., Rawat S., Ho K.L., Wagg C.S., Zhang L., Teoh H., Dyck J.E., Uddin G.M., Oudit G.Y., Mayoux E. (2018). Empagliflozin Increases Cardiac Energy Production in Diabetes. JACC: Basic Transl. Sci..

[217] Nielsen R., Møller N., Gormsen L.C., Tolbod L.P., Hansson N.H., Sorensen J., Harms H.J., Frøkiær J., Eiskjaer H., Jespersen N.R. (2019). Cardiovascular Effects of Treatment With the Ketone Body 3-Hydroxybutyrate in Chronic Heart Failure Patients. Circulation.

[218] Adingupu D.D., Göpel S.O., Grönros J., Behrendt M., Sotak M., Miliotis T., Dahlqvist U., Gan L.-M., Jönsson-Rylander A.-C. (2019). SGLT2 inhibition with empagliflozin improves coronary microvascular function and cardiac contractility in prediabetic ob/ob^−/−^ mice. Cardiovasc. Diabetol..

[219] Santos-Gallego C.G., Requena-Ibanez J.A., Antonio R.S., Ishikawa K., Watanabe S., Picatoste B., Flores E., Garcia-Ropero A., Sanz J., Hajjar R.J. (2019). Empagliflozin Ameliorates Adverse Left Ventricular Remodeling in Nondiabetic Heart Failure by Enhancing Myocardial Energetics. J. Am. Coll. Cardiol..

[220] Oh C.-M., Cho S., Jang J.-Y., Kim H., Chun S., Choi M., Park S., Ko Y.-G. (2019). Cardioprotective Potential of an SGLT2 Inhibitor Against Doxorubicin-Induced Heart Failure. Korean Circ. J..

[221] Shimazu T., Hirschey M.D., Newman J., He W., Shirakawa K., Le Moan N., Grueter C.A., Lim H., Saunders L.R., Stevens R.D. (2013). Suppression of Oxidative Stress by β-Hydroxybutyrate, an Endogenous Histone Deacetylase Inhibitor. Science.

[222] Youm Y.-H., Nguyen K.Y., Grant R.W., Goldberg E.L., Bodogai M., Kim D., D’Agostino D., Planavsky N., Lupfer C., Kanneganti T.-D. (2015). The ketone metabolite β-hydroxybutyrate blocks NLRP3 inflammasome–mediated inflammatory disease. Nat. Med..

[223] Kim S.R., Lee S.-G., Kim S.H., Kim J.H., Choi E., Cho W., Rim J., Hwang I., Lee C.J., Lee M. (2020). SGLT2 inhibition modulates NLRP3 inflammasome activity via ketones and insulin in diabetes with cardiovascular disease. Nat. Commun..

[224] Zhao X., Chen X., Zhang Y., George J., Cobbs A., Wang G., Li L., Emmett N. (2019). Kidney Injury Molecule-1 Is Upregulated in Renal Lipotoxicity and Mediates Palmitate-Induced Tubular Cell Injury and Inflammatory Response. Int. J. Mol. Sci..

[225] Little J.R., Spitzer J.J. (1971). Uptake of ketone bodies by dog kidney in vivo. Am. J. Physiol. Content.

[226] Fukao T., Song X.-Q., Mitchell G.A., Yamaguchi S., Sukegawa K., Or T., Kondo N. (1997). Enzymes of Ketone Body Utilization in Human Tissues: Protein and Messenger RNA Levels of Succinyl-Coenzyme A (CoA):3-Ketoacid CoA Transferase and Mitochondrial and Cytosolic Acetoacetyl-CoA Thiolases. Pediatric Res..

[227] Nosadini R., Trevisan R., Fioretto P., Semplicini A., Samà B., Velussi M., Da Campo G.L., Avogaro A., Vizzaccaro A., Donadon V. (1989). Kidney Hemodynamics After Ketone Body and Amino Acid Infusion in Normal and IDDM Subjects. Diabetes.

[228] Zhang D., Yang H., Kong X., Wang K., Mao X., Yan X., Wang Y., Liu S., Zhang X., Li J. (2011). Proteomics analysis reveals diabetic kidney as a ketogenic organ in type 2 diabetes. Am. J. Physiol. Metab..

[229] Verma S., McMurray J.J.V. (2018). SGLT2 inhibitors and mechanisms of cardiovascular benefit: A state-of-the-art review. Diabetologia.

[230] Chen L., LaRocque L.M., Efe O., Wang J., Sands J.M., Klein J.D. (2016). Effect of Dapagliflozin Treatment on Fluid and Electrolyte Balance in Diabetic Rats. Am. J. Med. Sci..

[231] Heerspink H.J.L., de Zeeuw D., Wie L., Leslie B., List J. (2013). Dapagliflozin a glucose-regulating drug with diuretic properties in subjects with type 2 diabetes. Diabetes Obes. Metab..

[232] Sha S., Polidori D., Heise T., Natarajan J., Farrell K., Wang S.-S., Sica D., Rothenberg P., Plum-Mörschel L. (2014). Effect of the sodium glucose co-transporter 2 inhibitor canagliflozin on plasma volume in patients with type 2 diabetes mellitus. Diabetes Obes. Metab..

[233] Griffin M., Rao V.S., Ivey-Miranda J., Fleming J., Mahoney D., Maulion C., Suda N., Siwakoti K., Ahmad T., Jacoby D. (2020). Empagliflozin in Heart Failure. Circulation.

[234] Karg M.V., Bosch A., Kannenkeril D., Striepe K., Ott C., Schneider M.P., Boemke-Zelch F., Linz P., Nagel A.M., Titze J. (2018). SGLT-2-inhibition with dapagliflozin reduces tissue sodium content: A randomised controlled trial. Cardiovasc. Diabetol..

[235] Packer M., Anker S.D., Butler J., Filippatos G., Ferreira J.P., Pocock S.J., Sattar N., Brueckmann M., Jamal W., Cotton D. (2021). Empagliflozin in Patients With Heart Failure, Reduced Ejection Fraction, and Volume Overload. J. Am. Coll. Cardiol..

[236] Yasui A., Lee G., Hirase T., Kaneko T., Kaspers S., Von Eynatten M., Okamura T. (2018). Empagliflozin Induces Transient Diuresis Without Changing Long-Term Overall Fluid Balance in Japanese Patients With Type 2 Diabetes. Diabetes Ther..

[237] Heise T., Jordan J., Wanner C., Heer M., Macha S., Mattheus M., Lund S.S., Woerle H.J., Broedl U.C. (2016). Pharmacodynamic Effects of Single and Multiple Doses of Empagliflozin in Patients With Type 2 Diabetes. Clin. Ther..

[238] Schnermann J. (1998). Juxtaglomerular cell complex in the regulation of renal salt excretion. Am. J. Physiol. Integr. Comp. Physiol..

[239] Francis G.S., Siegel R.M., Goldsmith S.R., Olivari M.T., Levine T.B., Cohn J.N. (1985). Acute Vasoconstrictor Response to Intravenous Furosemide in Patients with Chronic Congestive Heart Failure. Ann. Intern. Med..

[240] Škrtić M., Cherney D.Z. (2015). Sodium–glucose cotransporter-2 inhibition and the potential for renal protection in diabetic nephropathy. Curr. Opin. Nephrol. Hypertens..

[241] Hallow K.M., Helmlinger G., Greasley P.J., McMurray J.J.V., Boulton D.W. (2017). Why do SGLT2 inhibitors reduce heart failure hospitalization? A differential volume regulation hypothesis. Diabetes Obes. Metab..

[242] Jensen J., Omar M., Kistorp C., Tuxen C., Gustafsson I., Køber L., Gustafsson F., Faber J., Malik M.E., Fosbøl E.L. (2020). Effects of empagliflozin on estimated extracellular volume, estimated plasma volume, and measured glomerular filtration rate in patients with heart failure (Empire HF Renal): A prespecified substudy of a double-blind, randomised, placebo-controlled trial. Lancet Diabetes Endocrinol..

[243] Cohen N.D., Gutman S., Briganti E., Taylor A. (2019). Effects of empagliflozin treatment on cardiac function and structure in patients with type 2 diabetes: A cardiac magnetic resonance study. Intern. Med. J..

[244] Bosch A., Ott C., Jung S., Striepe K., Karg M.V., Kannenkeril D., Dienemann T., Schmieder R.E. (2019). How does empagliflozin improve arterial stiffness in patients with type 2 diabetes mellitus? Sub analysis of a clinical trial. Cardiovasc. Diabetol..

[245] Lunder M., Janić M., Japelj M., Juretič A., Janež A., Šabovič M. (2018). Empagliflozin on top of metformin treatment improves arterial function in patients with type 1 diabetes mellitus. Cardiovasc. Diabetol..

[246] Irace C., Cutruzzolà A., Parise M., Fiorentino R., Frazzetto M., Gnasso C., Casciaro F., Gnasso A. (2020). Effect of empagliflozin on brachial artery shear stress and endothelial function in subjects with type 2 diabetes: Results from an exploratory study. Diabetes Vasc. Dis. Res..

[247] Solini A., Giannini L., Seghieri M., Vitolo E., Taddei S., Ghiadoni L., Bruno R.M. (2017). Dapagliflozin acutely improves endothelial dysfunction, reduces aortic stiffness and renal resistive index in type 2 diabetic patients: A pilot study. Cardiovasc. Diabetol..

[248] Solini A., Seghieri M., Giannini L., Biancalana E., Parolini F., Rossi C., Dardano A., Taddei S., Ghiadoni L., Bruno R.M. (2019). The Effects of Dapagliflozin on Systemic and Renal Vascular Function Display an Epigenetic Signature. J. Clin. Endocrinol. Metab..

[249] Pfeifer M., Townsend R.R., Davies M.J., Vijapurkar U., Ren J. (2017). Effects of canagliflozin, a sodium glucose co-transporter 2 inhibitor, on blood pressure and markers of arterial stiffness in patients with type 2 diabetes mellitus: A post hoc analysis. Cardiovasc. Diabetol..

[250] Katakami N., Mita T., Yoshii H., Shiraiwa T., Yasuda T., Okada Y., Torimoto K., Umayahara Y., Kaneto H., Osonoi T. (2021). Effect of tofogliflozin on arterial stiffness in patients with type 2 diabetes: Prespecified sub-analysis of the prospective, randomized, open-label, parallel-group comparative UTOPIA trial. Cardiovasc. Diabetol..

[251] Aroor A.R., Das N.A., Carpenter A.J., Habibi J., Jia G., Ramirez-Perez F., Martinez-Lemus L., Manrique-Acevedo C.M., Hayden M.R., Duta C. (2018). Glycemic control by the SGLT2 inhibitor empagliflozin decreases aortic stiffness, renal resistivity index and kidney injury. Cardiovasc. Diabetol..

[252] Lee D.M., Battson M.L., Jarrell D.K., Hou S., Ecton K.E., Weir T.L., Gentile C.L. (2018). SGLT2 inhibition via dapagliflozin improves generalized vascular dysfunction and alters the gut microbiota in type 2 diabetic mice. Cardiovasc. Diabetol..

[253] Li H., Shin S.E., Seo M.S., An J.R., Choi I.-W., Jung W.-K., Firth A.L., Lee D.-S., Yim M.-J., Choi G. (2018). The anti-diabetic drug dapagliflozin induces vasodilation via activation of PKG and Kv channels. Life Sci..

[254] Striepe K., Jumar A., Ott C., Karg M.V., Schneider M.P., Kannenkeril D., Schmieder R.E. (2017). Effects of the Selective Sodium-Glucose Cotransporter 2 Inhibitor Empagliflozin on Vascular Function and Central Hemodynamics in Patients With Type 2 Diabetes Mellitus. Circulation.

[255] Weber T., Wassertheurer S., Rammer M., Haiden A., Hametner B., Eber B. (2012). Wave Reflections, Assessed With a Novel Method for Pulse Wave Separation, Are Associated With End-Organ Damage and Clinical Outcomes. Hypertension.

[256] Durante W., Behnammanesh G., Peyton K.J. (2021). Effects of Sodium-Glucose Co-Transporter 2 Inhibitors on Vascular Cell Function and Arterial Remodeling. Int. J. Mol. Sci..

[257] Bray J.J., Foster-Davies H., Stephens J.W. (2020). A systematic review examining the effects of sodium-glucose cotransporter-2 inhibitors (SGLT2is) on biomarkers of inflammation and oxidative stress. Diabetes Res. Clin. Pract..

[258] Nabrdalik-Leśniak D., Nabrdalik K., Sedlaczek K., Główczyński P., Kwiendacz H., Sawczyn T., Hajzler W., Drożdż K., Hendel M., Irlik K. (2021). Influence of SGLT2 Inhibitor Treatment on Urine Antioxidant Status in Type 2 Diabetic Patients: A Pilot Study. Oxidative Med. Cell. Longev..

[259] Hotamisligil G.S. (2006). Inflammation and metabolic disorders. Nature.

[260] Garvey W.T., Van Gaal L., Leiter L.A., Vijapurkar U., List J., Cuddihy R., Ren J., Davies M.J. (2018). Effects of canagliflozin versus glimepiride on adipokines and inflammatory biomarkers in type 2 diabetes. Metabolism.

[261] Benetti E., Mastrocola R., Vitarelli G., Cutrin J.C., Nigro D., Chiazza F., Mayoux E., Collino M., Fantozzi R. (2016). Empagliflozin Protects against Diet-Induced NLRP-3 Inflammasome Activation and Lipid Accumulation. J. Pharmacol. Exp. Ther..

[262] Xu L., Nagata N., Chen G., Nagashimada M., Zhuge F., Ni Y., Sakai Y., Kaneko S., Ota T. (2019). Empagliflozin reverses obesity and insulin resistance through fat browning and alternative macrophage activation in mice fed a high-fat diet. BMJ Open Diabetes Res. Care.

[263] Kusaka H., Koibuchi N., Hasegawa Y., Ogawa H., Kim-Mitsuyama S. (2016). Empagliflozin lessened cardiac injury and reduced visceral adipocyte hypertrophy in prediabetic rats with metabolic syndrome. Cardiovasc. Diabetol..

[264] Patel V.B., Shah S., Verma S., Oudit G.Y. (2017). Epicardial adipose tissue as a metabolic transducer: Role in heart failure and coronary artery disease. Heart Fail Rev..

[265] Salvatore T., Galiero R., Caturano A., Vetrano E., Rinaldi L., Coviello F., Di Martino A., Albanese G., Colantuoni S., Medicamento G. (2022). Dysregulated Epicardial Adipose Tissue as a Risk Factor and Potential Therapeutic Target of Heart Failure with Preserved Ejection Fraction in Diabetes. Biomolecules.

[266] Zibadi S., Cordova F., Slack E.H., Watson R.R., Larson D.F. (2011). Leptin’s regulation of obesity-induced cardiac extracellular matrix remodeling. Cardiovasc. Toxicol..

[267] Packer M. (2018). Do sodium-glucose co-transporter-2 inhibitors prevent heart failure with a preserved ejection fraction by counterbalancing the effects of leptin? A novel hypothesis. Diabetes Obes. Metab..

[268] Hao Z., Huang X., Shao H., Tian F. (2018). Effects of dapagliflozin on serum uric acid levels in hospitalized type 2 diabetic patients with inadequate glycemic control: A randomized controlled trial. Ther. Clin. Risk Manag..

[269] Braga T.T., Forni M.F., Correa-Costa M., Ramos R.N., Barbuto J.A.M., Branco P., Castoldi A., Hiyane M.I., Davanso M.R., Latz E. (2017). Soluble Uric Acid Activates the NLRP3 Inflammasome. Sci. Rep..

[270] Prattichizzo F., De Nigris V., Micheloni S., La Sala L., Ceriello A. (2018). Increases in circulating levels of ketone bodies and cardiovascular protection with SGLT2 inhibitors: Is low-grade inflammation the neglected component?. Diabetes Obes. Metab..

[271] Yaribeygi H., Atkin S.L., Butler A.E., Sahebkar A. (2018). Sodium–glucose cotransporter inhibitors and oxidative stress: An update. J. Cell. Physiol..

[272] Salvatore T., Caturano A., Galiero R., Di Martino A., Albanese G., Vetrano E., Sardu C., Marfella R., Rinaldi L., Sasso F.C. (2021). Cardiovascular Benefits from Gliflozins: Effects on Endothelial Function. Biomedicines.

[273] Tahara A., Kurosaki E., Yokono M., Yamajuku D., Kihara R., Hayashizaki Y., Takasu T., Imamura M., Li Q., Tomiyama H. (2013). Effects of SGLT2 selective inhibitor ipragliflozin on hyperglycemia, hyperlipidemia, hepatic steatosis, oxidative stress, inflammation, and obesity in type 2 diabetic mice. Eur. J. Pharmacol..

[274] Tahara A., Kurosaki E., Yokono M., Yamajuku D., Kihara R., Hayashizaki Y., Takasu T., Imamura M., Li Q., Tomiyama H. (2014). Effects of sodium-glucose cotransporter 2 selective inhibitor ipragliflozin on hyperglycaemia, oxidative stress, inflammation and liver injury in streptozotocin-induced type 1 diabetic rats. J. Pharm. Pharmacol..

[275] Salim H.M., Fukuda D., Yagi S., Soeki T., Shimabukuro M., Sata M. (2016). Glycemic Control with Ipragliflozin, a Novel Selective SGLT2 Inhibitor, Ameliorated Endothelial Dysfunction in Streptozotocin-Induced Diabetic Mouse. Front. Cardiovasc. Med..

[276] Oelze M., Kröller-Schön S., Welschof P., Jansen T., Hausding M., Mikhed Y., Stamm P., Mader M., Zinßius E., Agdauletova S. (2014). The Sodium-Glucose Co-Transporter 2 Inhibitor Empagliflozin Improves Diabetes-Induced Vascular Dysfunction in the Streptozotocin Diabetes Rat Model by Interfering with Oxidative Stress and Glucotoxicity. PLoS ONE.

[277] Steven S., Oelze M., Hanf A., Kröller-Schön S., Kashani F., Roohani S., Welschof P., Kopp M., Gödtel-Armbrust U., Xia N. (2017). The SGLT2 inhibitor empagliflozin improves the primary diabetic complications in ZDF rats. Redox Biol..

[278] Shigiyama F., Kumashiro N., Miyagi M., Ikehara K., Kanda E., Uchino H., Hirose T. (2017). Effectiveness of dapagliflozin on vascular endothelial function and glycemic control in patients with early-stage type 2 diabetes mellitus: DEFENCE study. Cardiovasc. Diabetol..

[279] Tanaka A., Shimabukuro M., Machii N., Teragawa H., Okada Y., Shima K.R., Takamura T., Taguchi I., Hisauchi I., Toyoda S. (2019). Effect of Empagliflozin on Endothelial Function in Patients With Type 2 Diabetes and Cardiovascular Disease: Results from the Multicenter, Randomized, Placebo-Controlled, Double-Blind EMBLEM Trial. Diabetes Care.

[280] Iannantuoni F., De Marañon A.M., Diaz-Morales N., Falcon R., Bañuls C., Abad-Jimenez Z., Victor V.M., Hernandez-Mijares A., Rovira-Llopis S. (2019). The SGLT2 Inhibitor Empagliflozin Ameliorates the Inflammatory Profile in Type 2 Diabetic Patients and Promotes an Antioxidant Response in Leukocytes. J. Clin. Med..

[281] Lee N., Heo Y.J., Choi S.-E., Jeon J.Y., Han S.J., Kim D.J., Kang Y., Lee K.W., Kim H.J. (2021). Anti-inflammatory Effects of Empagliflozin and Gemigliptin on LPS-Stimulated Macrophage via the IKK/NF-κB, MKK7/JNK, and JAK2/STAT1 Signalling Pathways. J. Immunol. Res..

[282] Heymans S., Hirsch E., Anker S.D., Aukrust P., Balligand J.-L., Cohen-Tervaert J.W., Drexler H., Filippatos G., Felix S.B., Gullestad L. (2009). Inflammation as a therapeutic target in heart failure? A scientific statement from the Translational Research Committee of the Heart Failure Association of the European Society of Cardiology. Eur. J. Heart Fail..

[283] Ye Y., Bajaj M., Yang H.-C., Perez-Polo J.R., Birnbaum Y. (2017). SGLT-2 Inhibition with Dapagliflozin Reduces the Activation of the Nlrp3/ASC Inflammasome and Attenuates the Development of Diabetic Cardiomyopathy in Mice with Type 2 Diabetes. Further Augmentation of the Effects with Saxagliptin, a DPP4 Inhibitor. Cardiovasc. Drugs Ther..

[284] Byrne N.J., Matsumura N., Maayah Z.H., Ferdaoussi M., Takahara S., Darwesh A.M., Levasseur J.L., Jahng J.W.S., Vos D., Parajuli N. (2020). Empagliflozin Blunts Worsening Cardiac Dysfunction Associated With Reduced NLRP3 (Nucleotide-Binding Domain-Like Receptor Protein 3) Inflammasome Activation in Heart Failure. Circ. Heart Fail..

[285] Koyani C.N., Plastira I., Sourij H., Hallström S., Schmidt A., Rainer P.P., Bugger H., Frank S., Malle E., von Lewinski D. (2020). Empagliflozin protects heart from inflammation and energy depletion via AMPK activation. Pharmacol. Res..

[286] Chen H., Tran D., Yang H.-C., Nylander S., Birnbaum Y., Ye Y. (2020). Dapagliflozin and Ticagrelor Have Additive Effects on the Attenuation of the Activation of the NLRP3 Inflammasome and the Progression of Diabetic Cardiomyopathy: An AMPK–mTOR Interplay. Cardiovasc. Drugs Ther..

[287] Kohlhaas M., Liu T., Knopp A., Zeller T., Ong M.F., Böhm M., O’Rourke B., Maack C. (2010). Elevated Cytosolic Na^+^ Increases Mitochondrial Formation of Reactive Oxygen Species in Failing Cardiac Myocytes. Circulation.

[288] Li C., Zhang J., Xue M., Li X., Han F., Liu X., Xu L., Lu Y., Cheng Y., Li T. (2019). SGLT2 inhibition with empagliflozin attenuates myocardial oxidative stress and fibrosis in diabetic mice heart. Cardiovasc. Diabetol..

[289] Hasan R., Lasker S., Hasan A., Zerin F., Zamila M., Chowdhury F.I., Nayan S.I., Rahman M., Khan F., Subhan N. (2020). Canagliflozin attenuates isoprenaline-induced cardiac oxidative stress by stimulating multiple antioxidant and anti-inflammatory signaling pathways. Sci. Rep..

[290] Lee T.-M., Chang N.-C., Lin S.-Z. (2017). Dapagliflozin, a selective SGLT2 Inhibitor, attenuated cardiac fibrosis by regulating the macrophage polarization via STAT3 signaling in infarcted rat hearts. Free Radic. Biol. Med..

[291] Lee H.-C., Shiou Y.-L., Jhuo S.-J., Chang C.-Y., Liu P.-L., Jhuang W.-J., Dai Z.-K., Chen W.-Y., Chen Y.-F., Lee A.-S. (2019). The sodium–glucose co-transporter 2 inhibitor empagliflozin attenuates cardiac fibrosis and improves ventricular hemodynamics in hypertensive heart failure rats. Cardiovasc. Diabetol..

[292] Kang S., Verma S., Hassanabad A.F., Teng G., Belke D.D., Dundas J.A., Guzzardi D.G., Svystonyuk D.A., Pattar S.S., Park D.S. (2019). Direct Effects of Empagliflozin on Extracellular Matrix Remodelling in Human Cardiac Myofibroblasts: Novel Translational Clues to Explain EMPA-REG OUTCOME Results. Can. J. Cardiol..

[293] Sun X., Han F., Lu Q., Li X., Ren D., Zhang J., Han Y., Xiang Y.K., Li J. (2020). Empagliflozin Ameliorates Obesity-Related Cardiac Dysfunction by Regulating Sestrin2-Mediated AMPK-mTOR Signaling and Redox Homeostasis in High-Fat Diet–Induced Obese Mice. Diabetes.

[294] Franssen C., Chen S., Unger A., Korkmaz H.I., De Keulenaer G.W., Tschöpe C., Leite-Moreira A.F., Musters R., Niessen H.W., Linke W.A. (2016). Myocardial Microvascular Inflammatory Endothelial Activation in Heart Failure With Preserved Ejection Fraction. JACC Heart Fail..

[295] Juni R.P., Kuster D.W., Goebel M., Helmes M., Musters R.J., van der Velden J., Koolwijk P., Paulus W.J., van Hinsbergh V.W. (2019). Cardiac Microvascular Endothelial Enhancement of Cardiomyocyte Function Is Impaired by Inflammation and Restored by Empagliflozin. JACC Basic Transl. Sci..

[296] Hamdani N., Bishu K.G., Von Frieling-Salewsky M., Redfield M.M., Linke W.A. (2012). Deranged myofilament phosphorylation and function in experimental heart failure with preserved ejection fraction. Cardiovasc. Res..

[297] Pabel S., Wagner S., Bollenberg H., Bengel P., Kovács A., Schach C., Tirilomis P., Mustroph J., Renner A., Gummert J. (2018). Empagliflozin directly improves diastolic function in human heart failure. Eur. J. Heart Fail..

[298] Xue M., Li T., Wang Y., Chang Y., Cheng Y., Lu Y., Liu X., Xu L., Li X., Yu X. (2019). Empagliflozin prevents cardiomyopathy via sGC-cGMP-PKG pathway in type 2 diabetes mice. Clin. Sci..

[299] Kolijn D., Pabel S., Tian Y., Lódi M., Herwig M., Carrizzo A., Zhazykbayeva S., Kovács Á., Fülöp G.., Falcão-Pires I. (2020). Empagliflozin improves endothelial and cardiomyocyte function in human heart failure with preserved ejection fraction via reduced pro-inflammatory-oxidative pathways and protein kinase Gα oxidation. Cardiovasc. Res..

[300] Yaribeygi H., Butler A.E., Atkin S.L., Katsiki N., Sahebkar A. (2018). Sodium–glucose cotransporter 2 inhibitors and inflammation in chronic kidney disease: Possible molecular pathways. J. Cell. Physiol..

[301] Ishibashi Y., Matsui T., Yamagishi S. (2015). Tofogliflozin, A Highly Selective Inhibitor of SGLT2 Blocks Proinflammatory and Proapoptotic Effects of Glucose Overload on Proximal Tubular Cells Partly by Suppressing Oxidative Stress Generation. Horm. Metab. Res..

[302] Xu J., Kitada M., Ogura Y., Liu H., Koya D. (2021). Dapagliflozin Restores Impaired Autophagy and Suppresses Inflammation in High Glucose-Treated HK-2 Cells. Cells.

[303] Das N.A., Carpenter A.J., Belenchia A., Aroor A.R., Noda M., Siebenlist U., Chandrasekar B., DeMarco V.G. (2019). Empagliflozin reduces high glucose-induced oxidative stress and miR-21-dependent TRAF3IP2 induction and RECK suppression, and inhibits human renal proximal tubular epithelial cell migration and epithelial-to-mesenchymal transition. Cell Signal..

[304] Satou R., Cypress M.W., Woods T.C., Katsurada A., Dugas C.M., Fonseca V.A., Navar L.G. (2020). Blockade of sodium-glucose cotransporter 2 suppresses high glucose-induced angiotensinogen augmentation in renal proximal tubular cells. Am. J. Physiol. Physiol..

[305] Birnbaum Y., Bajaj M., Yang H.-C., Ye Y. (2018). Combined SGLT2 and DPP4 Inhibition Reduces the Activation of the Nlrp3/ASC Inflammasome and Attenuates the Development of Diabetic Nephropathy in Mice with Type 2 Diabetes. Cardiovasc. Drugs Ther..

[306] Wu R., Liu X., Yin J., Wu H., Cai X., Wang N., Qian Y., Wang F. (2018). IL-6 receptor blockade ameliorates diabetic nephropathy via inhibiting inflammasome in mice. Metabolism.

[307] Hasan R., Lasker S., Hasan A., Zerin F., Zamila M., Parvez F., Rahman M., Khan F., Subhan N., Alam A. (2020). Canagliflozin ameliorates renal oxidative stress and inflammation by stimulating AMPK–Akt–eNOS pathway in the isoprenaline-induced oxidative stress model. Sci. Rep..

[308] Jaikumkao K., Pongchaidecha A., Chueakula N., Thongnak L., Wanchai K., Chatsudthipong V., Chattipakorn N., Lungkaphin A. (2018). Dapagliflozin, a sodium-glucose co-transporter-2 inhibitor, slows the progression of renal complications through the suppression of renal inflammation, endoplasmic reticulum stress and apoptosis in prediabetic rats. Diabetes Obes. Metab..

[309] Elkazzaz S.K., Khodeer D.M., El Fayoumi H.M., Moustafa Y.M. (2021). Role of sodium glucose cotransporter type 2 inhibitors dapagliflozin on diabetic nephropathy in rats; Inflammation, angiogenesis and apoptosis. Life Sci..

[310] Shiraki A., Kotooka N., Komoda H., Hirase T., Oyama J.-I., Node K. (2016). Pentraxin-3 regulates the inflammatory activity of macrophages. Biochem. Biophys. Rep..

[311] Huang F., Zhao Y., Wang Q., Hillebrands J.-L., Born J.V.D., Ji L., An T., Qin G. (2019). Dapagliflozin Attenuates Renal Tubulointerstitial Fibrosis Associated With Type 1 Diabetes by Regulating STAT1/TGFβ1 Signaling. Front. Endocrinol..

[312] Heerspink H.J.L., Perco P., Mulder S., Leierer J., Hansen M.K., Heinzel A., Mayer G. (2019). Canagliflozin reduces inflammation and fibrosis biomarkers: A potential mechanism of action for beneficial effects of SGLT2 inhibitors in diabetic kidney disease. Diabetologia.

[313] Mulder S., Hammarstedt A., Nagaraj S.B., Nair V., Ju W., Hedberg J., Greasley P.J., Eriksson J.W., Oscarsson J., Heerspink H.J.L. (2020). A metabolomics-based molecular pathway analysis of how the sodium-glucose co-transporter-2 inhibitor dapagliflozin may slow kidney function decline in patients with diabetes. Diabetes Obes. Metab..

[314] Chen Y., Liu Y., Dorn G.W. (2011). Mitochondrial Fusion is Essential for Organelle Function and Cardiac Homeostasis. Circ. Res..

[315] Ong S.-B., Subrayan S., Lim S.Y., Yellon D.M., Davidson S.M., Hausenloy D.J. (2010). Inhibiting Mitochondrial Fission Protects the Heart Against Ischemia/Reperfusion Injury. Circulation.

[316] Onishi M., Yamano K., Sato M., Matsuda N., Okamoto K. (2021). Molecular mechanisms and physiological functions of mitophagy. EMBO J..

[317] Durak A., Olgar Y., Degirmenci S., Akkus E., Tuncay E., Turan B. (2018). A SGLT2 inhibitor dapagliflozin suppresses prolonged ventricular-repolarization through augmentation of mitochondrial function in insulin-resistant metabolic syndrome rats. Cardiovasc. Diabetol..

[318] Zhou H., Wang S., Zhu P., Hu S., Chen Y., Ren J. (2017). Empagliflozin rescues diabetic myocardial microvascular injury via AMPK-mediated inhibition of mitochondrial fission. Redox Biol..

[319] Mizuno M., Kuno A., Yano T., Miki T., Oshima H., Sato T., Nakata K., Kimura Y., Tanno M., Miura T. (2018). Empagliflozin normalizes the size and number of mitochondria and prevents reduction in mitochondrial size after myocardial infarction in diabetic hearts. Physiol. Rep..

[320] Sciarretta S., Maejima Y., Zablocki D., Sadoshima J. (2018). The Role of Autophagy in the Heart. Annu. Rev. Physiol..

[321] Packer M. (2020). Autophagy stimulation and intracellular sodium reduction as mediators of the cardioprotective effect of sodium–glucose cotransporter 2 inhibitors. Eur. J. Heart Fail..

[322] Xu C., Wang W., Zhong J., Lei F., Xu N., Zhang Y., Xie W. (2018). Canagliflozin exerts anti-inflammatory effects by inhibiting intracellular glucose metabolism and promoting autophagy in immune cells. Biochem. Pharmacol..

[323] Aragón-Herrera A., Feijóo-Bandín S., Santiago M.O., Barral L., Campos-Toimil M., Gil-Longo J., Pereira T.M.C., García-Caballero T., Rodríguez-Segade S., Rodríguez J. (2019). Empagliflozin reduces the levels of CD36 and cardiotoxic lipids while improving autophagy in the hearts of Zucker diabetic fatty rats. Biochem. Pharmacol..

[324] Jiang K., Xu Y., Wang D., Chen F., Tu Z., Qian J., Xu S., Xu Y., Hwa J., Li J. (2021). Cardioprotective mechanism of SGLT2 inhibitor against myocardial infarction is through reduction of autosis. Protein Cell.

[325] Wang C.-Y., Chen C.-C., Lin M.-H., Su H.-T., Ho M.-Y., Yeh J.-K., Tsai M.-L., Hsieh I.-C., Wen M.-S. (2020). TLR9 Binding to Beclin 1 and Mitochondrial SIRT3 by a Sodium-Glucose Co-Transporter 2 Inhibitor Protects the Heart from Doxorubicin Toxicity. Biology.

[326] Zhan M., Brooks C., Liu F., Sun L., Dong Z. (2013). Mitochondrial dynamics: Regulatory mechanisms and emerging role in renal pathophysiology. Kidney Int..

[327] Takagi S., Li J., Takagaki Y., Kitada M., Nitta K., Takasu T., Kanasaki K., Koya D. (2018). Ipragliflozin improves mitochondrial abnormalities in renal tubules induced by a high-fat diet. J. Diabetes Investig..

[328] Lee Y.H., Kim S.H., Kang J.M., Heo J.H., Kim D.-J., Park S.H., Sung M., Kim J., Oh J., Yang D.H. (2019). Empagliflozin attenuates diabetic tubulopathy by improving mitochondrial fragmentation and autophagy. Am. J. Physiol. Physiol..

[329] Tang C., Livingston M.J., Liu Z., Dong Z. (2020). Autophagy in kidney homeostasis and disease. Nat. Rev. Nephrol..

[330] Umino H., Hasegawa K., Minakuchi H., Muraoka H., Kawaguchi T., Kanda T., Tokuyama H., Wakino S., Itoh H. (2018). High Basolateral Glucose Increases Sodium-Glucose Cotransporter 2 and Reduces Sirtuin-1 in Renal Tubules through Glucose Transporter-2 Detection. Sci. Rep..

[331] Packer M. (2020). Role of Impaired Nutrient and Oxygen Deprivation Signaling and Deficient Autophagic Flux in Diabetic CKD Development: Implications for Understanding the Effects of Sodium-Glucose Cotransporter 2-Inhibitors. J. Am. Soc. Nephrol..

[332] Korbut A.I., Taskaeva I.S., Bgatova N.P., Muraleva N.A., Orlov N.B., Dashkin M.V., Khotskina A., Zavyalov E.L., Konenkov V.I., Klein T. (2020). SGLT2 Inhibitor Empagliflozin and DPP4 Inhibitor Linagliptin Reactivate Glomerular Autophagy in db/db Mice, a Model of Type 2 Diabetes. Int. J. Mol. Sci..

[333] Sano M., Goto S. (2019). Possible Mechanism of Hematocrit Elevation by Sodium Glucose Cotransporter 2 Inhibitors and Associated Beneficial Renal and Cardiovascular Effects. Circulation.

[334] Sano M., Takei M., Shiraishi Y., Suzuki Y. (2016). Increased Hematocrit During Sodium-Glucose Cotransporter 2 Inhibitor Therapy Indicates Recovery of Tubulointerstitial Function in Diabetic Kidneys. J. Clin. Med. Res..

[335] Thiele K., Rau M., Hartmann N.K., Möllmann J., Jankowski J., Böhm M., Keszei A.P., Marx N., Lehrke M. (2021). Effects of empagliflozin on erythropoiesis in patients with type 2 diabetes: Data from a randomized, placebo-controlled study. Diabetes Obes. Metab..

[336] Haase V.H. (2006). Hypoxia-inducible factors in the kidney. Am. J. Physiol. Physiol..

[337] O’Neill J., Fasching A., Pihl L., Patinha D., Franzén S., Palm F. (2015). Acute SGLT inhibition normalizes O2 tension in the renal cortex but causes hypoxia in the renal medulla in anaesthetized control and diabetic rats. Am. J. Physiol. Physiol..

[338] Takaori K., Nakamura J., Yamamoto S., Nakata H., Sato Y., Takase M., Nameta M., Yamamoto T., Economides A., Kohno K. (2015). Severity and Frequency of Proximal Tubule Injury Determines Renal Prognosis. J. Am. Soc. Nephrol..

[339] Symeonidis A., Kouraklis-Symeonidis A., Psiroyiannis A., Leotsinidis M., Kyriazopoulou V., Vassilakos P., Vagenakis A., Zoumbos N. (2005). Inappropriately low erythropoietin response for the degree of anemia in patients with noninsulin-dependent diabetes mellitus. Ann. Hematol..

[340] Ghanim H., Abuaysheh S., Hejna J., Green K., Batra M., Makdissi A., Chaudhuri A., Dandona P. (2020). Dapagliflozin Suppresses Hepcidin And Increases Erythropoiesis. J. Clin. Endocrinol. Metab..

[341] Mazer C.D., Hare G.M., Connelly P.W., Gilbert R.E., Shehata N., Quan A., Teoh H., Leiter L.A., Zinman B., Jüni P. (2020). Effect of Empagliflozin on Erythropoietin Levels, Iron Stores, and Red Blood Cell Morphology in Patients with Type 2 Diabetes Mellitus and Coronary Artery Disease. Circulation.

[342] Hare G.M.T., Zhang Y., Chin K., Thai K., Jacobs E., Cazorla-Bak M.P., Nghiem L., Wilson D.F., Vinogradov S.A., Connelly K.A. (2021). Impact of sodium glucose linked cotransporter-2 inhibition on renal microvascular oxygen tension in a rodent model of diabetes mellitus. Physiol. Rep..

[343] Maruyama T., Takashima H., Oguma H., Nakamura Y., Ohno M., Utsunomiya K., Furukawa T., Tei R., Abe M. (2019). Canagliflozin Improves Erythropoiesis in Diabetes Patients with Anemia of Chronic Kidney Disease. Diabetes Technol. Ther..

[344] Oshima M., Neuen B.L., Jardine M.J., Bakris G., Edwards R., Levin A., Mahaffey K.W., Neal B., Pollock C., Rosenthal N. (2020). Effects of canagliflozin on anaemia in patients with type 2 diabetes and chronic kidney disease: A post-hoc analysis from the CREDENCE trial. Lancet Diabetes Endocrinol..

[345] Yanai H., Katsuyayama H. (2017). A Possible Mechanism for Renoprotective Effect of Sodium-Glucose Cotransporter 2 Inhibitor: Elevation of Erythropoietin Production. J. Clin. Med. Res..

[346] Verma S., Mazer C.D., Yan A.T., Mason T., Garg V., Teoh H., Zuo F., Quan A., Farkouh M.E., Fitchett D.H. (2019). Effect of Empagliflozin on Left Ventricular Mass in Patients With Type 2 Diabetes Mellitus and Coronary Artery Disease. Circulation.

[347] Fitchett D., Inzucchi S.E., Zinman B., Wanner C., Schumacher M., Schmoor C., Ohneberg K., Ofstad A.P., Salsali A., George J.T. (2021). Mediators of the improvement in heart failure outcomes with empagliflozin in the EMPA-REG OUTCOME trial. ESC Heart Fail..

